# Interphase-Resolved Performance in PA6/TiO_2_ Nanocomposite Fibers: Four-Phase Geometry Linking Structure to Mechanical and UV Protection

**DOI:** 10.3390/polym17182551

**Published:** 2025-09-21

**Authors:** Hailong Yu, Ping Liu, Xiaohuan Ji, Xiaoze Jiang, Bin Sun

**Affiliations:** 1 State Key Laboratory of Advanced Fiber Materials, College of Materials Science and Engineering, Donghua University, Shanghai 201620, China; 2 Shaoxing Huiqun New Material Technology Co., Ltd., Shaoxing 312000, China

**Keywords:** PA6/TiO_2_ fibers, polymer nanocomposite fibers, rigid amorphous fraction (RAF), interphase, synchrotron SAXS, Porod invariant, WAXS, Hermans orientation factor, non-isothermal crystallization, UPF

## Abstract

Melt-spun PA6/TiO_2_ fibers with TiO_2_ modified by silane coupling agents KH550 and KH570 at 0, 1.6, and 4 wt% provide a practical testbed to address three fiber-centric gaps: transferable interphase quantification, interphase-resolved indications of compatibility, and a reproducible kinetics–structure–property link. This work proposes, for the first time at fiber scale, a four-phase partition into crystal (c), crystal-adjacent rigid amorphous fraction (RAF-c), interfacial rigid amorphous fraction (RAF-i), and mobile amorphous fraction (MAF), and extracts an interfacial triad consisting of the specific interfacial area (S_v_), polymer-only RAF-i fraction expressed per composite volume (Γ_i_), and interphase thickness (t_i_) from SAXS invariants to establish a quantitative interphase-structure–property framework. A documented SAXS/DSC/WAXS workflow partitions the polymer into the above four components on a polymer-only basis. Upon filling, Γ_i_ increases while RAF-c decreases, leaving the total RAF approximately conserved. Under identical cooling, DSC shows the crystallization peak temperature is higher by 1.6–4.3 °C and has longer half-times, indicating enhanced heterogeneous nucleation together with growth are increasingly limited by interphase confinement. At 4 wt% loading, KH570-modified fibers versus KH550-modified fibers exhibit higher α-phase orientation (Hermans factor f(α): 0.697 vs. 0.414) but an ~89.4% lower α/γ ratio. At the macroscale, compared to pure (neat) PA6, 4 wt% KH550- and KH570-modified fibers show tenacity enhancements of ~9.5% and ~33.3%, with elongation decreased by ~31–68%. These trends reflect orientation-driven stiffening accompanied by a reduction in the mobile amorphous fraction and stronger interphase constraints on chain mobility. Knitted fabrics achieve a UV protection factor (UPF) of at least 50, whereas pure PA6 fabrics show only ~5.0, corresponding to ≥16-fold improvement. Taken together, the SAXS-derived descriptors (S_v_, Γ_i_, t_i_) provide transferable interphase quantification and, together with WAXS and DSC, yield a reproducible link from interfacial geometry to kinetics, structure, and properties, revealing two limiting regimes—orientation-dominated and phase-fraction-dominated.

## 1. Introduction

Polyamide-6 (PA6)/TiO_2_ nanocomposite fibers are compelling for advanced textiles, filtration, and protective garments because they can combine mechanical robustness, thermal stability, and ultraviolet (UV) shielding within a single platform. Beyond overall loading, macroscopic behavior reflects the combined effects of interfacial chemistry and interphase geometry, via constrained chain dynamics that influence non-isothermal crystallization, orientation during drawing, and long-term durability. Within semicrystalline polymers, the rigid amorphous fraction (RAF) provides a physically grounded descriptor for such constrained domains and has long been linked to crystallization pathways and morphology development [1,2,3,4,5,6]. This conceptualization aligns with long-standing SAXS/WAXS and crystallization practice in polyamides, including precedents on non-isothermal kinetics and X-ray-based crystallinity quantification [7,8,9]; and is also consistent with metastable-state interpretations of semicrystalline transitions [10,11,12]. Organic–inorganic hybrid fiber systems therefore provide an effective route to integrate functional synergy with interfacial regulation, and recent reviews have summarized hybridization and interface-engineering strategies for multifunctional fibers [13,14]. These studies underline the centrality of interfacial design, and complementary analyses of TiO_2_ nanocomposites emphasize that an enlarged interfacial area produces a significant polymer fraction with properties distinct from the bulk, yet reproducible quantification remains under development [15]. A systematic framework that resolves interphase geometry and connects it to mechanical and UV-protection performance is therefore needed to advance PA6/TiO_2_ fibers.

Recent works have expanded RAF analysis to diverse polymer systems, covering its temperature-dependent quantification [16], development under cold-crystallization conditions [17], and even its use as an indicator for polymer–polymer interactions [18]. These developments, however, remain rooted in the classical three-phase picture crystal(c), RAF, and MAF—which continues to provide powerful framework for semicrystalline polymers [1,2,5,8,19]. In nanocomposites, RAF can be apportioned by origin into RAF-c and RAF-i, enabling interface-resolved analysis of constrained domains without abandoning the three-phase foundation [20]. Four-phase RAF partitioning has been implemented in bulk/thin-film nanocomposites and linked to kinetic/dynamical readouts [18], yet its application to melt-spun fibers is technically challenging: invariant-based SAXS workflows require stringent procedures, and fiber anisotropy couples Porod terms with α/γ orientation. Precedents exist in thin films and electrospun mats [21,22], but fiber studies rarely adopt geometry-normalized and interphase-resolved descriptors that are transferable across processing conditions [23,24]. In parallel, the confined mechanical response of RAF has been directly quantified [25], and its thermal stability recently linked to lamellar thickening in nascent iPPs [26].

In melt-spun PA6/TiO_2_ fibers, high TiO_2_ loadings induce aggregation and weak interfaces, undermining strength and UV gains. Silane surface functionalization mitigates these drawbacks [27,28,29], and polymeric compatibilizers reported for PA6 can further reinforce the interface [30]. Recent PA6-fiber studies have mapped process–property windows, grafting-based thermal/UV stabilization, and interphase tailoring via macromolecular anchoring [31,32,33]. Yet fiber-relevant and reproducible descriptors that capture the geometry and continuity of the interfacial constrained layer under melt-spinning remain limited [15,34]. Against this backdrop, three gaps remain: (i) transferable fiber-scale quantification of the interphase-constrained amorphous fraction is scarce (downsizing amplifies interfacial area and yields fractions distinct from bulk [20,24]); (ii) routine XRD/WAXS, SAXS, DSC, and microscopy assess dispersion and compatibility but rarely provide interphase-resolved, geometry-normalized metrics for the geometry/redistribution of constrained amorphous material [35,36,37,38,39]; and (iii) a reproducible, in-one-study bridge linking non-isothermal crystallization kinetics [40,41,42,43], WAXS-derived orientation and polymorphism, and properties remains uncommon [3,23,44]. Recent studies further highlight these issues: interphase-modulated RAF effects were observed in PA66/MMT nanocomposites [45], strain-dependent RAF evolution was revealed under deformation [46]. Together with recent insights into RAF stability and confinement, these findings reinforce the relevance of addressing the three gaps identified here at the fiber scale.

Here, we address these gaps by extending the classical three-phase picture to a polymer-only four-phase partition (c, RAF-c, RAF-i, MAF). On this basis, SAXS Porod–Debye invariants after crystalline-peak subtraction provide the specific interfacial area (S_v_) [47,48,49], which, combined with DSC crystallinity, yields an interfacial-allocation descriptor (Γ_i_); together, they are summarized by an effective thickness (t_i_) as geometry-normalized readouts at the fiber scale. These descriptors are then linked to non-isothermal crystallization kinetics, WAXS-derived α/γ phase fractions and Hermans orientation [50], and non-isothermal kinetics are evaluated via Nakamura global fitting with complementary Kissinger/KAS closures consistent with ICTAC recommendations [40,41,42,43,51]. Complementary characterizations (mechanical, rheological, SEM, UPF [52], and qualitative coarse-grained MD) and sensitivity checks are summarized in Methods/SI.

To rationalize the material selection, PA6 was chosen as a representative semi-crystalline fiber matrix for its spinnability and established structure–property framework; TiO_2_ nanoparticles were incorporated for their dual role as UV-shielding fillers and nucleating agents; KH550 (amine-functional) and KH570 (methacrylate-functional) silanes were selected to contrast interfacial chemistries. The novelty of this work lies in introducing interphase-resolved four-phase geometry at the fiber scale and establishing transferable SAXS-derived descriptors (S_v_, Γ_i_, t_i_) that reproducibly link interphase geometry to crystallization kinetics, microstructural orientation, and macroscopic performance. The overarching objective is to demonstrate that such geometry provides a unifying and transferable basis for understanding and tailoring mechanical and UV-protection properties in PA6/TiO_2_ nanocomposite fibers.

## 2. Materials and Methods

### 2.1. Materials and Surface Modification

Polyamide 6 (PA6, grade M2400; Xinhui Meida Chemical Co., Ltd., Jiangmen, China) from a single production lot was used for all experiments. According to the manufacturer’s specification for grade M2400, this is a fiber-grade, unfilled PA6 suitable for melt processing. Pellets were pre-crystallized in a vacuum rotary drum oven (JM-500ZDGX, Shanghai Jinma Electric Co., Ltd., Shanghai, China) at 90 °C for 18 h, followed by vacuum drying at 135 °C for 18 h immediately prior to processing to minimize residual moisture. Titanium dioxide (TiO_2_, TA-300, Fuji Titanium, Osaka, Japan; mean particle size—0.613 μm) was dried at 80 °C for 12 h and stored under nitrogen. The silane coupling agents γ-aminopropyltriethoxysilane (KH550, ≥98%) and γ-methacryloxypropyltrimethoxysilane (KH570, ≥98%) were purchased from Shanghai Chemical Reagent Co., Ltd., Shanghai, China and tetrabutyl titanate (TBOT, ≥99%) was supplied by Sinopharm Chemical Reagent Co., Ltd., Shanghai, China. The mass ratio of silane to TiO_2_ was fixed at 5 wt%. Antioxidant Irganox 1010 (BASF SE, Ludwigshafen, Germany, 0.1 wt%), release agent HLL-1 (Covestro, Leverkusen, Germany, 0.05 wt%), and flow modifier Lotader AX8900 (Arkema S.A., 0.2 wt%, Colombes, France) were used as processing aids. Solvents included anhydrous ethanol and n-hexane (both ≥99%, Sinopharm Chemical Reagent, Shanghai, China), as well as deionized water prepared in-house.

For the surface modification of TiO_2_, 8.00 kg of pre-dried TiO_2_ powder was dispersed in 25.0 L anhydrous ethanol in a 50 L glass reactor, and 1.60 L TBOT was added. The mixture was stirred at room temperature for 60 min and left standing for 24 h. After filtration and washing with ethanol and deionized water, the product was vacuum dried at 80 °C for 12 h to yield TBOT-coated TiO_2_ (p-TiO_2_). For silane modification, 2.32 kg KH550 or KH570 was dissolved in 25.0 L ethanol, and 5.6 L deionized water was slowly added (silane: water: ethanol ≈ 1:2:8). The solution was hydrolyzed at pH 5.5 for 1 h under stirring, then p-TiO_2_ was gradually added. The suspension was heated to 70 °C and stirred for 4 h, then filtered, washed, and vacuum dried at 80 °C for 12 h to obtain silane-modified TiO_2_ nanoparticles. Although the silane-to-TiO_2_ ratio during modification was fixed at 5 wt%, only DLS was performed on the modified powders. The effective surface coupling was then assessed after compounding—FTIR-ATR on PA6/TiO_2_ masterbatches and Raman/DMA/melt rheology on composite specimens—and is reported in the Results.

**Scheme 1:**
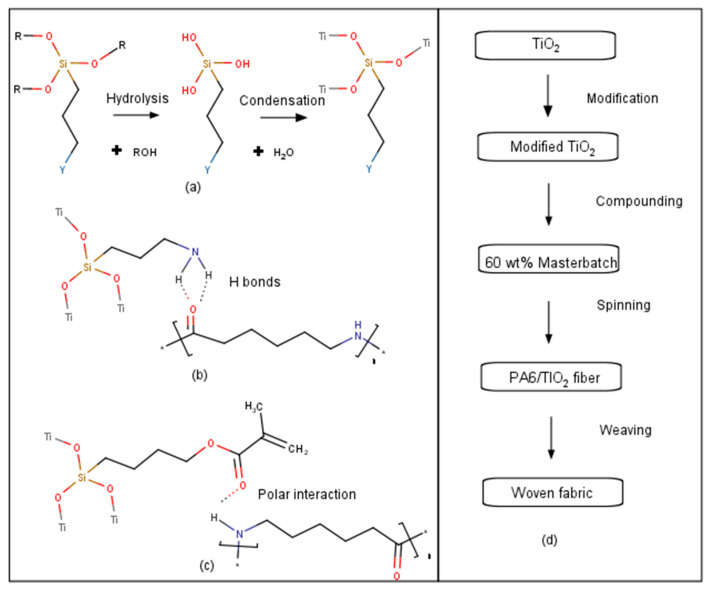
Schematic illustration of (**a**) hydrolysis and condensation of silane coupling agents on TiO_2_ surface, (**b**) interfacial hydrogen bonding between KH550 and PA6, (**c**) polar interaction between KH570 and PA6, and (**d**) processing route from surface modification to masterbatch compounding, fiber spinning, and fabric weaving.

The overall modification mechanism, interfacial interactions, and subsequent processing strategy are summarized schematically in Scheme 1.

In panel (a), the hydrolysis and condensation of silane coupling agents on TiO_2_ are illustrated. The alkoxy groups (–OR) of KH550 or KH570 undergo hydrolysis to form silanol groups (–Si–OH), which then condense with surface hydroxyl groups on TiO_2_ to yield covalent Si–O–Ti bonds. This reaction ensures stable anchoring of the silane molecules on the oxide surface, while the organic functional groups (–NH_2_ for KH550, –C(=O)O–C=C– for KH570) remain exposed for subsequent interaction with the polymer matrix.

In panel (b), the interfacial hydrogen bonding between KH550 and PA6 is depicted. The amino end group (–NH_2_) of KH550 acts as a hydrogen-bond donor, forming bifurcated hydrogen bonds with the carbonyl oxygen atoms of PA6 chains. This dual hydrogen-bonding interaction enhances interfacial compatibility and immobilizes the amorphous PA6 fraction adjacent to TiO_2_, strengthening stress transfer across the filler–matrix interface.

In panel (c), the polar interaction between KH570 and PA6 is shown. Unlike KH550, KH570 does not contain an amino group, but instead a methacrylate ester moiety. The ester carbonyl oxygen can serve as a weak hydrogen-bond acceptor with PA6 amide N–H groups. This results in less pronounced interfacial binding compared to KH550, which is reflected in weaker confinement of the amorphous PA6 phase.

Finally, panel (d) presents the overall processing route from surface modification of TiO_2_ powders, through masterbatch compounding at high filler loadings (up to 60 wt%), to dilution with neat PA6 and subsequent melt spinning at ≤6 wt% TiO_2_, followed by weaving into fabrics. This schematic highlights how surface chemistry at the nanoparticle level is integrated into fiber-scale processing, ultimately linking interfacial interactions to macroscopic textile structures.

### 2.2. Fiber Preparation and Processing

Dried PA6 and silane-modified TiO_2_ powders were premixed in specific mass ratios, together with Irganox 1010 (0.1 wt%) and HLL-1 (0.05 wt%) as processing aids. The mixtures were dried at 120 °C under vacuum for 12 h prior to compounding.

Masterbatches containing 50 wt% and 60 wt% TiO_2_ were prepared using a CTE20 twin-screw extruder (Nanjing KEYA, Nanjing, China; screw diameter 20 mm, L/D = 25). The barrel temperature profile was set at 235–248 °C, and the screw speed was 350 rpm. The melt was filtered through 100-mesh and 200-mesh screens, extruded through a 2 mm die, water-cooled, pelletized (2–3 mm), and vacuum dried at 110 °C for 6 h. The masterbatches were designated as “PA6–50%TiO_2_-KH550/570” and “PA6–60%TiO_2_-KH550/570”.

For fiber spinning, the masterbatches were diluted with pure (neat) PA6 to obtain different TiO_2_ contents. The dried blends were processed on an ABE-25 single-screw extruder (operated at ~60% channel fill, 100 rpm). The temperature zones were set at 280–300 °C, with a 36-hole spinneret (0.15 mm diameter), dual-layer filters (50- and 200-mesh), and side air cooling. The as-spun fibers were collected at 800–1000 m/min and immediately drawn and heat-set using a UDY+DT stretching machine (hot plate 60 °C, hot roll 120 °C to a fixed draw ratio (DR) 3.0. The resulting yarns were named according to the coupling agent and their TiO_2_ content. The samples are named and referenced as shown in Table 1 for direct comparison of TiO_2_ content, coupling agent, and draw ratio.

The KH550 series (0, 1.6, and 4.0 wt%) was selected to resolve concentration-dependent interfacial effects, given its stronger hydrogen-bonding affinity with PA6. In contrast, KH570 was included at 4.0 wt% as a representative high-loading case to highlight differences between coupling agents; this loading was chosen to maximize contrast in interfacial behavior, rather than to form a full concentration series.

### 2.3. Characterization Techniques

#### 2.3.1. Dynamic Light Scattering (DLS)

Particle size distributions of silane-modified TiO_2_ powders (KH550 or KH570) were measured using a Nano ZS90 instrument (Malvern Panalytical Ltd., Malvern, UK) equipped with a 633 nm laser at a scattering angle of 90°. Powders were dispersed in absolute ethanol and ultrasonicated for 10 min prior to measurement at 25 ± 0.1 °C. Z-average diameter and polydispersity index (PDI) were obtained from intensity-weighted distributions (*n* = 3 independent runs) using the instrument software and in-house scripts, and results are reported as mean ± standard error of the mean (SE). A PDI value below 0.2 was considered indicative of monodispersity, following Bhattacharjee [53].

#### 2.3.2. Filter Pressure Value (FPV) Testing

**Figure 1:**
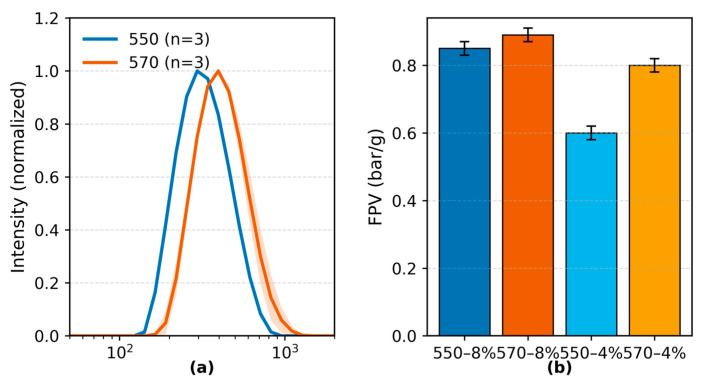
(**a**) DLS particle size distributions of KH550- and KH570-modified TiO_2_ powders in ethanol. (**b**) Filtration pressure values (FPV) of PA6/TiO_2_ masterbatches at 4 wt% and 8 wt% TiO_2_.

The filter pressure value was determined according to BS EN 13900-5:2005 [39] using standard melt-filtration equipment (Zibo Fangchen Masterbatch Factory, Zibo, China), without deviations from the prescribed test and calculation procedures. PA6/TiO_2_ masterbatches were diluted to the target TiO_2_ contents prior to testing [35]. FPV testing was conducted once per condition, consistent with the TBOOT (test based on one trial) approach previously validated for comparable masterbatch systems [54,55], where repeatability is ensured by the standardized procedure and narrow process tolerances. Reported values are therefore represent single standardized measurements (see Figure 1b in the Results section).

#### 2.3.3. Fourier-Transform Infrared (FTIR) Spectroscopy

FTIR spectra were recorded using a Nicolet iS10 spectrometer (Thermo Fisher Scientific Inc., Waltham, MA, USA) in attenuated total reflectance (ATR) mode over 400–4000 cm^−1^, with 32 scans averaged at 4 cm^−1^ resolution. Samples were PA6 masterbatches containing unmodified or silane-modified TiO_2_ (60 wt% TiO_2_ in a PA6 carrier), dried at 80 °C for 12 h prior to measurement. Spectra were normalized to the 0–1 range and analyzed between 4000 and 650 cm^−1^. Difference spectra were obtained by least-squares scaling outside 930–1100 cm^−1^ to isolate silane–TiO_2_-related absorptions. Each spectrum represents the averaged response of multiple scans, and replicate acquisitions under identical conditions yielded consistent peak positions and relative intensities, confirming reproducibility (see representative spectra in the Results section).

#### 2.3.4. Raman Spectroscopy

**Figure 2:**
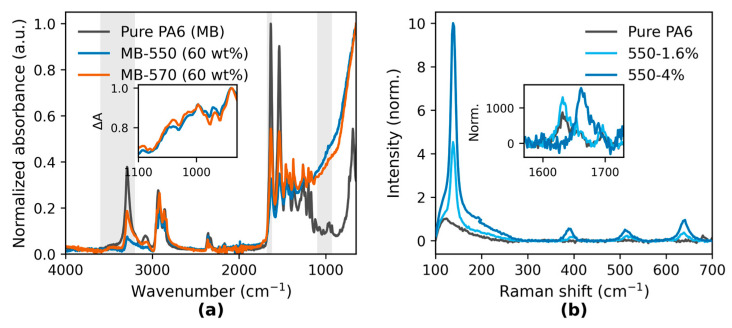
(**a**) FTIR spectra of pure PA6 and silane-treated TiO_2_ masterbatches, highlighting O–H/N–H, amide I, and Si–O–Ti/Si–O–Si bands. (**b**) Raman spectra of pure PA6 and selected composites; insets show difference and magnified amide I region.

Raman spectra were collected on a Renishaw inVia Reflex confocal Raman microscope (Renishaw plc., Wotton-under-Edge, UK) using 532 nm laser excitation (50× objective, spectral range 100–3200 cm^−1^). Injection-molded specimens (pure PA6 and composites containing 1.6 and 4.0 wt% TiO_2_ with KH550 modification) were polished for reproducible optical contact. Spectra were acquired with an integration time of 10 s, with three accumulations, at a laser power of ~5 mW measured at the sample surface. Data were normalized to the 95th percentile intensity over 100–700 cm^−1^. The amide I region (1600–1700 cm^−1^) was additionally inspected by magnified plots to compare peak profiles among samples. eCharacteristic bands agree with literature assignments for PA6 and TiO_2_, supporting the reliability of the measurements, while the representative spectra are provided in the Results section (Figure 2).

#### 2.3.5. Scanning Electron Microscopy (SEM) and Energy-Dispersive X-Ray Spectroscopy (EDS)

Fiber microstructure and TiO_2_ dispersion were examined by SEM (JSM-5600LV, JEOL Ltd., Tokyo, Japan) operated at 1–10 kV accelerating voltage. Cross-sections of melt-spun PA6/TiO_2_ fibers were prepared by cryofracture in liquid nitrogen and sputter-coated with a thin Au layer. Elemental mapping and semi-quantitative analysis of Ti and O were performed using an attached EDS detector under identical imaging conditions. For each condition, at least multiple regions were examined, and characteristic morphologies and Ti/O distributions were found to be consistent across replicates, ensuring reproducibility. Representative SEM/EDS micrographs from related melt-spun fibers are provided in the Supporting Information (Figure S1).

#### 2.3.6. Differential Scanning Calorimetry (DSC)

**Figure 3:**
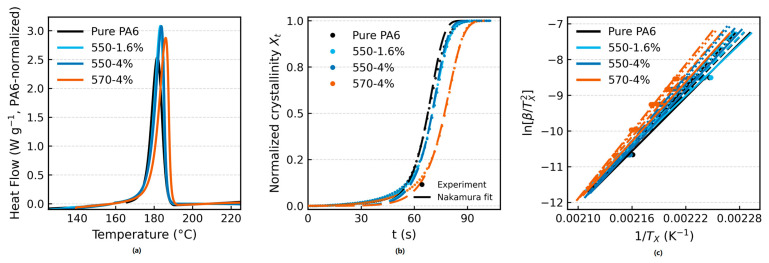
(**a**) Non-isothermal DSC cooling curves of pure PA6 and PA6/TiO_2_ fibers. (**b**) Relative crystallinity as a function of time with Nakamura fits. (**c**) Isoconversional KAS analysis at multiple conversions and cooling rates.

DSC is performed on a Pyris-1 (PerkinElmer Inc., Waltham, MA, USA) using ~5–10 mg specimens sealed in standard aluminum pans. Cooling scans are conducted at β = 5, 10, 20, 30, and 40 °C·min^−1^ under nitrogen. Heat-flow signals are blank-corrected for TiO_2_ and normalized to PA6 mass (W g^−1^). Representative cooling curves are shown in the Results section (Figure 3a) for pure PA6 and PA6/TiO_2_ fibers (0, 1.6, and 4 wt% KH550; 4 wt% KH570) at 20 °C·min^−1^.

Relative crystallinity X_t_(t) is obtained by integrating the exotherm between onset and endset determined by the tangent-intercept method, followed by 0–1 normalization. Representative plots are presented in the Results section (Figure 3b),where symbols indicate experimental data and dashed lines for Nakamura [42] fits based on the isothermal-equivalent time θ *, performed over X = 0.20–0.60, with activation energies fixed from independent isoconversional analysis.

Apparent activation energies are evaluated by Kissinger [40] using peak temperatures T_p_ (linear regression of ln [β/T_p_^2^] versus 1/T_p_) and by the isoconversional KAS [41,51,56] method at fixed conversions X = 0.30, 0.40, 0.50, and 0.55. Representative regressions are presented in the Results section (Figure 3c), where ln[β/T_x_^2^] versus 1/T_x_ plots at different conversion levels and cooling rates, where the slopes were used to obtain Ea(X). Temperatures in regressions are expressed in Kelvin. Parameters are reported as mean ± standard deviation; uncertainties for Kissinger/KAS follow linear-regression statistics, and uncertainties for the Nakamura fit are derived from the covariance of the global model [43].

Supplementary Information provides the full DSC cooling curves (Figure S3), per-conversion KAS regressions (Figure S4), single-point Kissinger regressions (Figure S5), and a parameter/uncertainty summary (Table S1). Each DSC cooling experiment was performed once per sample per rate. This follows common practice in polymer crystallization studies, where instrument stability and standardized protocols provide reproducible results. Reproducibility is further supported by the consistency between Kissinger and KAS kinetic analyses, and uncertainties are quantified from regression statistics rather than replicate runs.

#### 2.3.7. Synchrotron Small- and Wide-Angle X-Ray Scattering (SAXS/WAXS)

Unless otherwise noted, all phase fractions φ—φ_c_, φ_RAF-c_, φ_RAF-i_, φ_MAF_, φ_A_—are reported on a polymer-only basis (normalized to the polymer internal volume). The interfacial volume Γ_i_ is reported per composite volume as Γ_i_ = (1 − φ_if_)·φ_RAF-i_, where φ_if_ is the filler volume fraction. This convention is used consistently throughout all figures and tables.

SAXS experiments were conducted at the BL16B1 beamline of the Shanghai Synchrotron Radiation Facility (SSRF), Shanghai, China (λ = 0.124 nm) [57]. Fiber bundles were mounted on aluminum frames and equilibrated at 25 °C and 45% RH prior to measurement. Ag behenate was used for calibration. Raw images were reduced in pyFAI [58] with solid-angle and polarization corrections and exposure-scaled air subtraction, followed by azimuthal averaging to obtain I(q) (0.02–0.50 Å^−1^).

**Figure 4:**
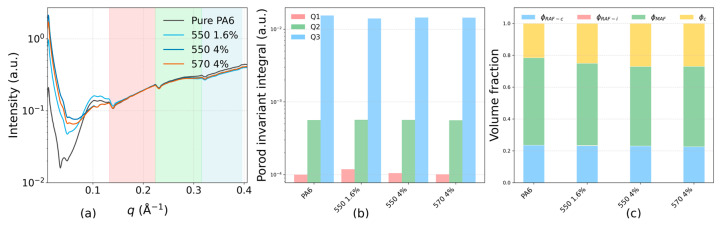
(**a**) SAXS intensity profiles of pure PA6 and selected nanocomposites. (**b**) Porod invariant integrals over defined q-regions. (**c**) Phase fractions resolved into φ_c_, φ_RAF−c_, φ_RAF−i_, and φ_MAF_.

Representative SAXS profiles were obtained for pure PA6, KH550-modified fibers with 1.6 and 4 wt% TiO_2_, and KH570-modified fibers with 4 wt% TiO_2_. Shaded regions (Q1: 0.08–0.14 Å^−1^, Q2: 0.14–0.23 Å^−1^, Q3: 0.23–0.50 Å^−1^) were integrated to obtain Porod invariants. These data are presented in the Results section (Figure 4a,b). Quantitative analysis used the Porod constant B and invariant Q: the specific interfacial area was obtained from S_v_ = 2π^2^ B/Q [47,48,49]. Polymer-internal phase fractions were resolved into four components—crystal (φ_c_), rigid amorphous fraction (φ_RAF–c_ + φ_RAF–i_), and mobile amorphous fraction (φ_MAF_)—normalized to the polymer internal volume [3]. The interface fraction was defined as Γ_i_ = φ_RAF–i_·(1–φ_f_), and the effective interfacial-layer thickness as t_i_ = Γ_i_/S_v_, enabling interface-resolved metrics [51,59].

Bragg peaks above q ≈ 0.14 Å^−1^ were masked/fitted and removed; the Porod invariant was integrated from the residuals over 0.02–0.50 Å^−1^, and the high-q Porod plateau was estimated on 0.32–0.40 Å^−1^. Each sample was measured once per condition, consistent with beamline practice where instrument stability and standardized reduction protocols ensure reproducibility; repeatability was supported by internal consistency between Porod-based invariants and DSC-derived crystallinity.

Details of masking, baseline handling, and residual-based Q integration for SAXS are provided in the SI (Figure S6; Table S2).

WAXS CeO_2_ was used for q/geometry calibration. After masking, solid-angle and polarization corrections, and exposure-scaled air subtraction, diffraction images were binned onto a q–χ grid [58]. Hermans orientation factors were evaluated from narrow q-bands centered at the PA6 α(200) (~1.717 Å^−1^) and γ(200/020) (~2.776 Å^−1^) reflections. For each band, intensities across Δq were summed (main analysis) to preserve peak-height information, and the normalized I(χ) was converted to f following the Hermans formalism and modern implementations [50,60]. Azimuthal sectors affected by instrumentation (−180° to −160°, 0° to 20°) were excluded. For γ, notch masks were applied near TiO_2_ lines and a fixed narrow Δq = 0.010–0.012 Å^−1^ was used. The azimuthal zero was fixed at 90° based on an 85–95° scan.

The α/γ crystalline phase ratio and the representative γ-phase crystallite size Dγ^(rep)^ were obtained from the integrated peak areas and Scherrer-type analysis, respectively, for pure PA6 and TiO_2_-filled fibers (550-1.6%, 550-4%, 570-4%). Each sample was measured once per condition, consistent with synchrotron WAXS practice, where high beam stability and standardized reduction pipelines ensure reproducibility; repeatability was supported by consistent trends across α/γ ratios, crystallite sizes, and Hermans orientation factors.

**Figure 5:**
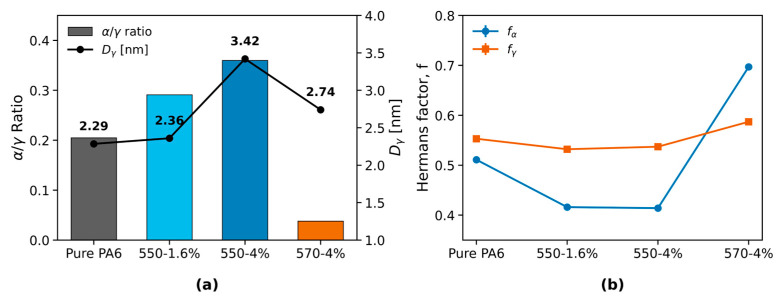
(**a**) α/γ crystalline phase ratio and γ-phase crystallite size. (**b**) Hermans orientation factors for α- and γ-phases.

Error bars represent standard deviations estimated from Monte-Carlo resampling (*N* = 300) over band center/width and noise injection, as presented in the Results section (Figure 5). Robustness checks using band-wise means (instead of sums), alternative sector exclusions, and baseline options, as well as representative patterns and azimuthal profiles, are provided in Figures S8–S10.

A targeted robustness analysis of the α(200) Hermans factor under single-ring, excluded-sector settings is summarized in Table S3.

#### 2.3.8. Dynamic Mechanical Analysis (DMA)

**Figure 6:**
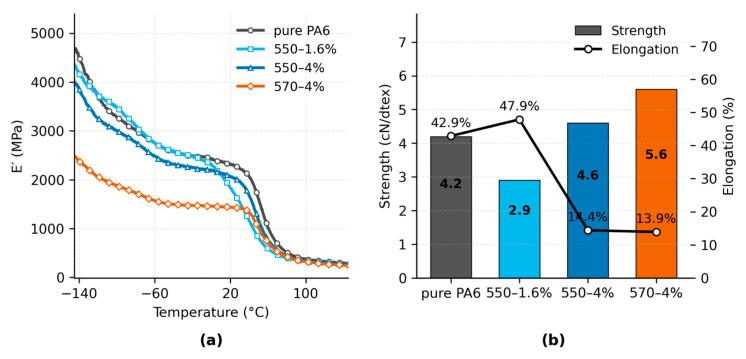
(**a**) Storage modulus as a function of temperature. (**b**) Tensile strength and elongation at break of pure PA6 and nanocomposite fibers.

DMA was conducted on a Q800 analyzer (TA Instruments, New Castle, DE, USA) in single-cantilever mode using injection-molded bars (35 × 10 × 2 mm). Temperature sweeps were run from −150 °C to 150 °C at 3 °C·min^−1^ and 1 Hz frequency after conditioning at 25 °C. Representative storage modulus curves were obtained; the results are presented in the Results section (Figure 6a). Each condition was tested once, consistent with DMA practice where instrument stability and standardized thermal protocols ensure reproducibility.

#### 2.3.9. Rotational Rheology

**Figure 7:**
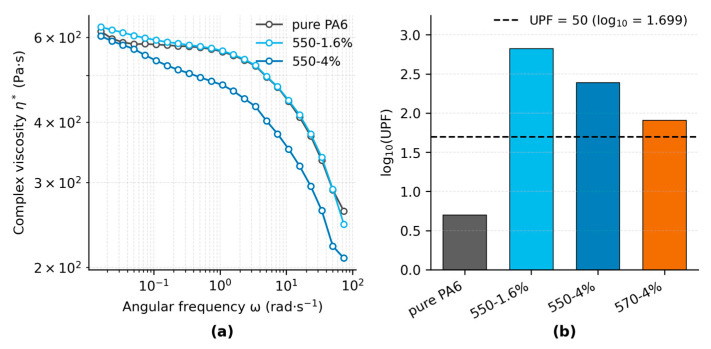
(**a**) Complex viscosity as a function of angular frequency at 230 °C. (**b**) Ultraviolet protection reported as log_10_(UPF) of plain-woven fabrics from pure PA6 and nanocomposite fibers.

Rotational rheology was performed on a Haake MARS rheometer (Thermo Fisher Scientific GmbH, Karlsruhe, Germany) with 20 mm in diameter, 1 mm thick disk specimens at 230 °C under nitrogen. Complex viscosity η*(ω) curves were obtained from frequency sweeps, as presented in the Results section (Figure 7a). Each sample was measured once per condition, consistent with rheology practice where instrument stability and standardized protocols ensure reproducibility.

#### 2.3.10. Mechanical Testing

Tensile properties of multifilament yarns were measured using an XL-2 instrument (Shanghai Xinfang Instrument Co., Ltd., Shanghai, China) according to the standard test method. At least 5 specimens per condition were tested, and results are reported as mean ± SD. Numerical labels correspond to average tensile strength values, while percentages denote mean elongation at break, as presented in the Results section (Figure 6b). Linear densities (dtex) are reported in Table S4.

#### 2.3.11. Ultraviolet Protection Factor (UPF)

Plain-woven fabric swatches (10 × 10 cm) were conditioned at 25 °C and measured on a fabric ultraviolet transmittance analyzer equipped with an integrating sphere (UV1000F, Labsphere Inc., North Sutton, NH, USA). Spectra were recorded over 250–450 nm; UPF was computed from the 290–400 nm region in accordance with EN 13758-1 [4] and AATCC TM183 [52], using the standard solar irradiance profile in EN 13758-1:2002. Each specimen was measured at multiple positions using a 3 × 3 grid across the swatch (*n* = 4–5 per specimen), and results are reported as the mean UPF together with the 95% one-sided lower confidence bound (UPF_LCB_). UPF measurements reflect fabric-level UV protection of plain-woven textiles prepared from the fibers. For visualization, log_10_(UPF) (dimensionless) is used, and samples with UPF ≥ 50 are denoted as 50+. UVA (315–400 nm) and UVB (290–315 nm) transmittance statistics are provided in the Supplementary Information.

#### 2.3.12. Molecular Dynamics (MD) Simulations

A two-stage, two-resolution coarse-grained (CG) workflow is employed to compare how silane coupling affects PA6–TiO_2_ crystallization. First, atomistic PA6 oligomers are mapped to Martini beads [61,62] (four beads per repeat unit) and equilibrated in GROMACS 2023 (Royal Institute of Technology, Stockholm, Sweden) [63] to yield equilibrium CG melt trajectories via polymerization simulation [64], from which chain statistics were computed. Equilibrated CG melt trajectories were remapped to a one-bead-per-repeat PA6 (CGPA) model and transferred to LAMMPS version 3 Mar 2020 (Sandia National Laboratories, Albuquerque, NM, USA) [65] for production runs. Two TiO_2_ surface models representing KH550-anchored and KH570-passivated interfaces were implemented [28,29,66]. At the Martini level, standard Martini parameters are used. In the CGPA model, bonded terms (bonds/angles) are obtained by Boltzmann inversion of distributions from the Martini trajectories [67,68,69,70], whereas non-bonded interactions are fitted to interaction-energy–distance data generated by ORCA 5.0.3 (Max Planck Institute for Coal Research, Mülheim an der Ruhr, Germany) [71] DLPNO-CCSD(T) [72]/aug-cc-pVTZ [73] scans of two PA6 repeat-unit fragments (inputs prepared by Multiwfn 3.8 (Beijing Kein Research Center for Natural Sciences, Beijing, China) [74]), and are implemented in LAMMPS as lj96/cut. Production runs follow an anneal–cool schedule consistent with processing; simulated heat-flow–temperature curves derived from energy–temperature traces are used to extract crystallization peak temperatures for comparison between the two surfaces. Representative snapshots and the simulated heat-flow traces for both surfaces are provided in the Supplementary Information (Figure S11). The simulations are used qualitatively to contextualize experimental trends rather than to assign quantitative causality. Simulations with independent initial configurations yielded consistent equilibrium descriptors, confirming reproducibility.

#### 2.3.13. Data Integration and Correlation Analysis

**Figure 8:**
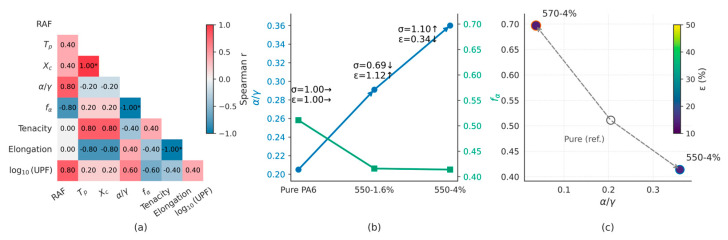
(**a**) Correlation map of interface, kinetics, structure, and performance descriptors. (**b**) Evolution of α/γ phase fraction and Hermans orientation factor f(α) in the KH550 series. (**c**) Structure–performance map linking f(α), α/γ, and mechanical/UV outcomes. Asterisks indicate statistically significant correlations based on Spearman tests (*p* < 0.05).

Eight parameters were selected to represent different levels: interphase (RAF), kinetics (T_p_), structure (X_c_, α/γ, f(α)), and performance (tenacity σ, elongation ε, and log_10_(UPF)). Units and definitions are listed in Table S2c. Spearman correlation coefficients were computed (n = 4 samples) using Python’s SciPy 1.12 (Python Software Foundation, Wilmington, DE, USA), with significance treated as exploratory due to sample size. Heatmaps visualizing correlation strength and sign are presented in the Results section (Figure 8a). The plots of α/γ ratios and Hermans orientation factors were plotted against TiO_2_ loading in the KH550 series are presented in the Results section (Figure 8b), with tensile strength and elongation annotated near each point for context. Structural descriptors (α/γ, f(α)) against performance indicators are provided in the Results section (Figure 8c), where marker size scales with σ and color with ε. Dashed arrows denote the shift from neat PA6 to nanocomposites. The geometric drivers S_v_, t_i_, and Γ_i_ are reported separately in Table S2c and discussed in Section 3.4.

#### 2.3.14. Generative AI Disclosure

ChatGPT (OpenAI, San Francisco, CA, USA; accessed June and July 2025) was used for minor language polishing and to assist in drafting and refining code snippets for data processing and visualization. No data or figures were generated by AI. All AI-assisted outputs were reviewed by the authors.

## 3. Results

### 3.1. Nanoparticle Dispersion and Interfacial Chemistry

Anatase TiO_2_ (TA-300) was surface-modified with KH550 or KH570 silane coupling agents to enhance nanoparticle dispersion and interfacial compatibility in the PA6 matrix. KH550 (aminosilane) introduces –NH_2_ groups capable of forming hydrogen bonds with the amide groups of PA6, thereby promoting strong interfacial adhesion, whereas KH570 (methacryloxy silane) offers weaker polar interactions, serving as a comparative modifier. This dual choice enables systematic evaluation of how interfacial chemistry governs dispersion, crystallization, and performance. The resulting powders and PA6 masterbatches were examined by DLS, FPV testing, FTIR, Raman spectroscopy, and SEM/EDS (Figure S1, Supporting Information). These complementary techniques yielded both quantitative and qualitative insights into particle size distribution, melt processability, chemical bonding at the polymer–filler interface, and spatial distribution of TiO_2_ in the matrix. Representative SEM/EDS micrographs from related melt-spun PA6/TiO_2_ fibers corroborated the dispersion and elemental distribution trends inferred from DLS, FPV, and spectroscopic analyses.

#### 3.1.1. TiO_2_ Dispersion and Interfacial Modification

Dynamic light scattering (Figure 1a) showed monomodal particle size distributions with narrow peaks for both KH550- and KH570-modified TiO_2_ powders, indicating uniform dispersion. The Z-average diameters were 359.6 nm for KH550-modified and 455.0 nm for KH570-modified TiO_2_, with corresponding PDIs of 0.120 and 0.115 (n = 3). Both PDI values were below the 0.2 threshold for monodispersity [53]. The slightly larger size of KH570 powders arises from looser silane coverage and minor re-agglomeration in ethanol, which did not affect dispersion in the final fibers, as confirmed by FPV and SEM/EDS.

FPV testing of PA6/TiO_2_ masterbatches at 4 wt% and 8 wt% filler loadings yielded values within the acceptable limits defined by BS EN 13900-5:2005 [39]. For KH550-modified TiO_2_, FPVs were 0.60 ± 0.02 bar g^−1^ (4 wt%) and 0.85 ± 0.02 bar g^−1^ (8 wt%); for KH570-modified TiO_2_, values were 0.80 ± 0.02 bar g^−1^ (4 wt%) and 0.89 ± 0.02 bar g^−1^ (8 wt%). All tested samples met the melt-filtration criteria for both low and high filler contents.

SEM images of representative melt-spun PA6/TiO_2_ fibers (Figure S1, Supporting Information) showed that, at 1.8 wt% TiO_2_, particles were uniformly distributed within the matrix, with core–shell-like features of approximately 200–300 nm. At 4.0 wt%, the particle density increased and occasional local adjacency was observed, but without formation of continuous agglomerated domains, as confirmed by higher-magnification SEM (Figure S1b). EDS mapping confirmed Ti and O signals in particle-rich zones, consistent with the nominal TiO_2_ contents. Additional low-magnification SEM and EDS mapping (Figure S12) further confirm fibrous continuity and TiO_2_ distribution consistent with nominal loadings, supporting effective but not perfect dispersion at higher filler contents.

#### 3.1.2. Chemical Interactions

The FTIR spectra of KH550-modified and KH570-modified TiO_2_ masterbatches (60 wt% TiO_2_ in PA6) exhibited additional absorption bands in the 930–1100 cm^−1^ region, which were not present in pure PA6 (Figure 2a, shaded area). The normalized absorbance in this region was higher for MB-570 than for MB-550. The inset difference spectra show positive deviations for both modified samples relative to pure PA6 in this range, with a slightly larger deviation for MB-570. A small shift in the amide I band (~1650 cm^−1^) towards lower wavenumbers was observed for both modified samples.

The Raman spectra (Figure 2b) were measured from injection-molded specimens prepared from the same masterbatch formulations but with different TiO_2_ loadings: KH550 series (0, 1.6, and 4 wt%) and KH570 series (4 wt%). Enhanced features in the low-frequency region (100–700 cm^−1^) were observed for all TiO_2_-containing samples compared with pure PA6. The inset focusing on the amide I region (1600–1700 cm^−1^) shows increased normalized peak intensities for the modified samples, with the 550-4 wt% specimen exhibiting the highest intensity and a slight shift in the peak position to lower wavenumbers relative to pure PA6. The full Raman spectra are provided in Figure S2 (Supplementary Information).

These spectral differences will be further considered in relation to rigid amorphous fraction (RAF) formation and crystallization behavior in Section 3.2.

### 3.2. Multi-Scale Structure and Crystallization Behavior

#### 3.2.1. Non-Isothermal Crystallization Kinetics: Avrami–Nakamura and Isoconversional Analyses

The crystallization kinetics of pure PA6 and PA6/TiO_2_ fiber composites were quantified using a θ*-based Nakamura framework (with activation energies fixed from independent isoconversional analysis) together with an isoconversional KAS approach.

Figure 3a shows the non-isothermal DSC cooling curves at 20 °C·min^−1^. All samples exhibit a single, well-defined exothermic peak. The peak crystallization temperature (T_p_) increases from 181.68 °C for pure PA6 to 183.23 °C (1.6 wt% TiO_2_–KH550), 183.74 °C (4 wt% TiO_2_–KH550), and 185.97 °C (4 wt% TiO_2_–KH570). The full width at half maximum (FWHM) narrows from 6.31 °C (pure PA6) to 5.43–5.83 °C for the composites. The crystallization enthalpy (ΔHc), normalized per gram of PA6 after TiO_2_ blank correction, is 55.10 J·g^−1^ for pure PA6 and 60.67–61.08 J·g^−1^ for the composites.

Figure 3b presents the evolution of relative crystallinity (X_t_) as a function of time at 20 °C·min^−1^. Using the Nakamura model with isothermal-equivalent time (θ*), global fits were performed over 0.20 ≤ X_t_ ≤ 0.60 with E fixed from the independent isoconversional analysis and rate-specific K_0_*. The fitted Avrami-type exponents were n = 3.147 (pure PA6), 2.871 (1.6 wt% TiO_2_–KH550), 2.762 (4 wt% TiO_2_–KH550), and 2.360 (4 wt% TiO_2_–KH570), with R^2^ = 0.864–0.925 across formulations. Despite the stricter cross-rate normalization, the rate scaling remained internally consistent: regression of ln K_0_* versus ln β gave αˆ ≈ 2.10–2.12 with R^2^ ≥ 0.989, and the predicted curves closely tracked the experimental X_t_(t) within the fit window. Uncertainty quantified by nonparametric bootstrap (B = 200) yielded 95% confidence intervals of 3.019–3.273, 2.691–3.107, 2.633–2.900, and 2.277–2.446 for the four samples, respectively; leave-one-rate validation gave nˉ = 3.144, 2.893, 2.765, and 2.356 (Table S6).

Figure 3c summarizes the isoconversional KAS (multi-point) analysis at X = 0.30, 0.40, 0.50, and 0.55 using cooling rates of 5–40 °C·min^−1^. Within this window, the apparent activation energy E_a_(X) shows weak conversion dependence; formulation-level medians are 208.2 kJ·mol^−1^ (pure PA6), 202.9 kJ·mol^−1^ (1.6 wt% TiO_2_–KH550), 229.6 kJ·mol^−1^ (4 wt% TiO_2_–KH550), and 237.4 kJ·mol^−1^ (4 wt% TiO_2_–KH570). Complementary single-point Kissinger regressions are provided in Figure S5, with full-rate DSC curves and per-conversion KAS fits in Figures S3 and S4, and parameter uncertainties (95% confidence intervals from bootstrap/leave-one-out) summarized in Table S6.

#### 3.2.2. Rigid Amorphous Fraction (RAF) Quantification

SAXS intensity profiles for four samples—Pure PA6, 550 1.6% (KH-550, 1.6 wt% TiO_2_), 550 4% (KH-550, 4 wt% TiO_2_), and 570 4% (KH-570, 4 wt% TiO_2_)—are shown in Figure 4a. All TiO_2_-filled samples exhibit higher scattering intensity in the low-q range (0.08–0.14 Å^−1^) than Pure PA6. In the mid-q range (0.14–0.23 Å^−1^), the four curves are generally comparable, whereas in the high-q range (0.23–0.50 Å^−1^) the TiO_2_-filled samples show slightly lower intensities than pure PA6.

As presented in Figure 4b, Porod invariant integration shows that Q1 (low-q) is consistently higher for all TiO_2_-filled samples than for Pure PA6. Q2 (mid-q) remains similar across the four samples, while Q3 (high-q) is lower for the TiO_2_-filled samples. The total Porod invariant (Q_total_) is comparable among all samples, indicating that the redistribution of scattering intensity occurs mainly between the low- and high-q regions.

Using DSC-derived crystalline fractions together with SAXS Porod-invariant analysis, we quantified the phase fractions on a polymer-only basis with a fixed pipeline (Figure 4c): first, the interfacial rigid amorphous fraction (φ_RAF-i_) was obtained by the low-q difference method, ΔQ_1_ = Q_1_, Sample − Q_1_, (pure PA6 constrained to φ_RAF-i_ = 0); second, φ_RAF-i_ was held fixed while the Q23 band was used to partition the crystal-adjacent RAF (φ_RAF-c_) and the mobile amorphous fraction (φ_MAF_). Point estimates and 95% bootstrap CIs are compiled in SI Table S2 (panels b and e); the Porod-invariant q-bands used are listed in Table S2a; the low-q baselines and increments that determine φ_RAF-i_ are in Table S2d; and interfacial metrics (S_v_, Γ_i_, t_i_) are given in Table S2c. Relative to pure PA6, all TiO_2_-filled samples show a non-zero, formulation-dependent φ_RAF-i_ and a compensating decrease in φ_MAF_, whereas φc (DSC) and φ_RAF-c_ remain within a similar range. Among the three filled formulations, the total constrained fraction normalized by amorphous content, RAF-tot/φA = (φ_RAF-c_ + φ_RAF-i_)/φ_A_, lies within a narrow band and is statistically equivalent under a ±0.01 TOST margin (Figure S7b), while pure PA6 exhibits the expected lower baseline; formulation-level differences in φ_RAF-i_ are visualized with 95% CIs in Figure S7a. This partition aligns with literature that distinguishes crystal-induced and interfacial rigid fractions [20].

#### 3.2.3. WAXS Analysis of Polymorphs and Orientation

The α/γ crystalline phase ratio (Figure 5a) was 0.205 for pure PA6 and increased to 0.291 for the 550-1.6% TiO_2_ fiber and 0.360 for the 550-4% TiO_2_ fiber. In contrast, the 570-4% TiO_2_ fiber exhibited a sharply reduced value of 0.038. The γ-phase crystallite size, Dγ^(rep)^, varied from 2.28 to 3.42 nm across the samples, with the largest value observed for the 550-4% TiO_2_ fiber and the smallest for pure PA6 (peak deconvolution shown in Figure S9).

For the Hermans orientation factors (Figure 5b), f(α) spans a wider range (0.41–0.70) compared with f(γ) (0.53–0.59). The highest α-phase orientation was observed in the 570-4 wt% TiO_2_ fiber (f(α) = 0.697), whereas the lowest occurred in the 550-4 wt% TiO_2_ fiber (f(α) = 0.414). By contrast, the γ-phase orientation exhibits only minor differences among the four samples (0.532–0.587). For 570-4 wt%, the α/γ ratio reaches the lowest value, while the Hermans factor of the α(200) reflection remains high and numerically stable (methodological validation in Figure S10, and extraction robustness is summarized in Figure S8). Sensitivity analyses (band width/center and noise injection; Table S3) confirm that the high f(α) observed for 570-4 wt% is robust against WAXS processing choices.

### 3.3. Macroscopic Functional and Mechanical Performance

#### 3.3.1. Stiffness and Strength

Figure 6a shows the temperature dependence of the storage modulus (*E′*) for pure PA6 and PA6/TiO_2_ fibers with different silane modifications. Across the measured range from –150 to 120 °C, all TiO_2_-filled samples maintained higher *E′* values than pure PA6, with the 550-4% sample exhibiting the highest modulus over most of the range. At low temperatures (<–50 °C), *E′* values were in the range of 4000–5000 MPa, while in the intermediate region (–50 °C to 50 °C) the modulus decreased progressively with increasing temperature. The 570-4% sample consistently showed lower *E′* values compared to other filled samples. The glass transition region was indicated by a rapid modulus drop between approximately 40 °C and 60 °C, consistent with *Tg* values determined from tan δ measurements.

Figure 6b summarizes the tensile properties measured at room temperature. Tensile strength ranged from 2.9 cN/dtex for the 550-1.6% sample to 5.6 cN/dtex for the 570-4% sample. Pure PA6 exhibited a strength of 4.2 cN/dtex, while the 550-4% sample reached 4.6 cN/dtex. Elongation at break varied inversely with strength, from 47.9% for 550-1.6% to 13.9% for 570-4%. Pure PA6 showed 42.9% elongation, higher than all samples except 550-1.6%. Overall, high-modulus samples tended to exhibit reduced elongation.

#### 3.3.2. Flowability and UV Protection

The complex viscosity (η*) curves of pure PA6 and TiO_2_-filled fibers (550-1.6% and 550-4%) are presented in Figure 7a. All samples exhibit typical shear-thinning behavior, where η* remains nearly constant at low angular frequencies and decreases progressively with increasing ω, consistent with typical shear-thinning behavior reported for polymer melts [75,76]. At ω = 100 rad·s^−1^, the η* values are approximately 4.10 × 10^2^ Pa·s for pure PA6, 4.14 × 10^2^ Pa·s for the 550-1.6% fiber, and 3.24 × 10^2^ Pa·s for the 550-4% fiber. The differences between samples become more pronounced at higher frequencies, with the 550-4% fiber showing the steepest reduction in viscosity, indicating weakened interfacial restriction and enhanced chain mobility at short time scales.

Figure 7b shows that all TiO_2_-filled fabrics reach UPF 50+, whereas pure PA6 provides only minimal protection. Pure PA6 (UPF = 5.02; UPF_LCB_95_% = 4.78; n = 4) is far below the threshold. Among filled samples, 550-1.6% (666.33; 636.86; n = 5) shows the highest protection, followed by 550-4% (246.61; 224.28; n = 4). 570-4% (81.30; 77.41; n = 5) is the lowest among the filled samples yet still exceeds the threshold. The ranking is consistent with UVB transmittance—0.05% (550-1.6%), 0.27% (550-4%), and 1.12% (570-4%)—whereas UVA varies less; full UVA/UVB statistics are listed in Table S5.

### 3.4. Interface–Structure–Performance Coupling

The Spearman correlation heatmap summarizes monotonic associations among interface (RAF), kinetics (T_p_), structure (X_c_, α/γ, f(α)), and performance (tenacity σ, elongation ε, log_10_(UPF)). On a polymer-only basis, X_c_ and T_p_ are strongly positively associated (r_s_ = 1.00). Phase composition and orientation are orthogonal in this set: α/γ and f(α) vary in opposite directions across the four specimens. Tenacity is positively associated with T_p_ and X_c_, whereas elongation shows the opposite tendencies. The log_10_(UPF) metric shows positive associations with RAF and α/γ and weaker associations with T_p_/X_c_ and f(α).

In the KH550 series (0, 1.6, and 4 wt% TiO_2_), α/γ increases from 0.21 to 0.29 and 0.36, whereas the Hermans factor of the α phase decreases from 0.511 to 0.416 and 0.414. The mechanical responses are non-monotonic: σ is 4.2, 2.9, and 4.6 cN/dtex, and ε is 42.9%, 47.9%, and 14.4%, respectively. The sequence denotes the series evolution; no optimality is implied.

At the same loading (4 wt% TiO_2_), KH570 shows a higher f(α) (0.697) and a lower α/γ (0.038) than KH550 (f(α) 0.414, α/γ 0.360). Among the three points displayed, KH570 exhibits the highest σ. Marker size encodes σ and color encodes ε; dashed arrows indicate the shift from the reference (Pure PA6).

## 4. Discussion

Building on the integrated dataset, we rationalize how interfacial chemistry restructures the constrained amorphous fraction (RAF) spatially, how such constraints couple to non-isothermal crystallization and phase orientation, and how these structural states relate to mechanical, rheological, and UV protection metrics, thereby providing transferable interphase quantification and interphase-resolved readouts of dispersion and compatibility. We emphasize mechanism-centric contrasts across silane types and loadings rather than optimality claims. Within this processing window and on a polymer-only basis, two equivalent routes emerge on the common outcome space: a geometry path (KH550 concentration series) and a chemistry-mediated friction path (4 wt% KH570 and KH550), whose endpoints are compactly visualized in Figure 8b,c, closing a reproducible kinetics–structure–property loop.

### 4.1. Role of Interfacial Chemistry in RAF Redistribution

Spectroscopic and dispersion evidence supports enhanced polymer–filler interactions with silane coupling agents. FTIR and Raman spectra show characteristic Si–O–C/Si–O–Si (and Ti–O–Si shoulder) features consistent with covalent grafting on TiO_2_ surfaces (Figure 2), in line with prior reports on silane-functionalized titania [27,36]. Complementary DLS, FPV, and SEM analyses demonstrate improved particle dispersion (Figure 1; Figure S1). On a polymer-only basis, SAXS-invariant analysis reveals that the total rigid amorphous fraction (RAF) remains approximately conserved, while its spatial allocation shifts from crystal-adjacent (φ_RAF-c_) to filler-proximal (φ_RAF-i_) zones (Figure 4c; Table S2b). This redistribution highlights that interfacial chemistry primarily relocates constrained amorphous domains rather than enlarging their overall amount, consistent with the established three-phase view of semicrystalline polymers [1,2,5,19,20,59,77].

To rationalize how a minor φ_RAF-i_ exerts measurable influence, we transform it into system-level descriptors. The interfacial RAF volume is defined as Γ_i_ = φ_RAF-i_^(poly-only)^·(1 − φ_f_), i.e., the portion of the entire composite volume occupied by polymer in the interfacial RAF. Dividing Γ_i_ by the specific interfacial area S_v_ yields an effective interfacial thickness t_i_ = Γ_i_/S_v_. Here, S_v_ is obtained from SAXS via the Porod constant and invariant (S_v_ = 2π^2^B/Q) (Methods; Figure 4a,b and Figure S6; Table S2a) [47,48,49]. The resulting t_i_ values are sub-nanometric (see Table S2c) and are treated as an effective descriptor rather than an absolute physical thickness; thin but spatially extensive interfacial skins can disproportionately affect chain mobility and non-isothermal crystallization. Such interfacial skins are known to impose disproportionate constraints on chain mobility and to modulate non-isothermal crystallization in semicrystalline polymers [9,51,66].

Along the KH550 concentration series (0, 1.6, and 4 wt%), the system evolves from an orientation-dominated regime—characterized by higher S_v_ and thinner t_i_—towards a phase-fraction-dominated regime at higher loading, where S_v_ decreases and t_i_ thickens (Table S2c). At comparable interfacial descriptors (4 wt% KH550 vs. 4 wt% KH570), the downstream kinetic–orientation responses diverge, suggesting that distinct coupling pathways are activated depending on the silane chemistry rather than interfacial area/skin thickness alone [28,29].

We therefore interpret the observed small-in-volume yet surface-abundant RAF-i through the (S_v_, t_i_, Γ_i_) triad, consistent with the interphase perspective in polymer nanocomposites [34,77,78].

At similar specific interfacial area S_v_ but distinct effective thickness t_i_ at 4 wt% loading, the outcomes diverge by chemistry: KH570 (passivated/low-friction) follows an orientation-dominated route (higher f(α) with lower α/γ), whereas KH550 (anchored/high-friction) follows a phase-fraction-dominated route (higher α/γ with lower f(α)) [30,79]. These contrasts do not require changing the total constrained fraction; they reflect RAF redistribution under comparable S_v_ and chemistry-dependent t_i_ and Γ_i_.

### 4.2. Influence of Interfacial RAF on Non-Isothermal Kinetics and Orientation

Increasing the interfacial RAF volume (Γ_i_) is associated with systematic shifts in non-isothermal crystallization metrics [42]: the supercooling ΔT_p_ decreases (higher T_p_) while −log_10_(t_1_/_2_) decreases (i.e., t_1_/_2_ lengthens) across formulations (Figure 3a,b). Partial Spearman correlations controlling TiO_2_ wt% and silane type support these associations (Table S5a: ρ(ΔT_p_, Γ_i_) < 0; ρ(−log_10_t_1_/_2_, Γ_i_) < 0). Taken together, these trends are consistent with an interfacial-constraint scenario [59] in which an ultrathin, high-coverage interfacial constrained layer provides earlier effective nucleation sites (reduced ΔT_p_) while constraining growth/transport (longer t_1_/_2_) in the Xt = 0.20–0.60 window used for Nakamura fitting [9,40,41,43] (Figure 3b; Table S1). Nakamura global fits (fixed Ea from isoconversional closure) show rate-dependent K* shifts with n varying modestly within uncertainty across formulations (Table S1), indicating changes in the overall time scale rather than a wholesale change in growth dimensionality.

WAXS reveals that phase composition (α/γ) and orientation (f(α) at α(200)) are orthogonal descriptors: a lower α/γ can coexist with a higher f(α)(Figure 5a,b). The Hermans factor is robust to q-band width, band-center, and noise injection (Table S3; Figures S8–S10), ensuring that observed differences reflect genuine orientation contrasts rather than processing artifacts [60]. Partial correlations indicate a negative association between f(α) and Γ_i_ (filled + pure set), but a positive association between f(α) and the interfacial thickness t_i_ (filled subset) (Table S5a). This pattern is consistent with two coupling pathways: orientation-dominated skins (large S_v_, small t_i_) correlate with higher f(α) and lower α/γ, whereas phase-fraction-dominated skins (smaller S_v_, larger t_i_) correlate with higher α/γ and lower f(α).

Consistent with 4.1, partial Spearman correlations (controlling TiO_2_ wt% and silane type) show that larger Γ_i_ associates with smaller ΔTp (higher T_p_) and longer t_1/2_ (Table S5a; Figure 3a,b). Along the KH550 series, S_v_ decreases while t_i_ increases, with Γ_i_ varying modestly within the small-n uncertainty (Table S2c); α/γ rises whereas f(α) falls, consistent with a shift toward the phase-fraction-dominated side. We refrain from causal claims beyond the reported statistics and use these outcome labels to summarize directionality.

### 4.3. Structural States and Their Relation to Macroscopic Performance

The interfacial descriptors identified above—α-phase orientation (f(α), interfacial RAF volume (Γ_i_), interfacial coverage (S_v_), and the mobile amorphous fraction (MAF)—provide a common coordinate system to interpret stiffness, strength/ductility, viscoelasticity, and UV protection.

Across filled formulations, the storage modulus E′ increases relative to pure PA6 (Figure 6a), consistent with additional interfacial constraints on segmental mobility. Within the same structural coordinate, tensile strength tracks with f(α) and Γ_i_, whereas elongation at break decreases in line with reduced MAF (Figure 6b) [80,81,82]. Partial Spearman correlations (controlling TiO_2_ wt% and silane type) and descriptive ΔR^2^ confirm that f(α) and Γ_i_ retain independent explanatory power beyond composition (Table S5a,b). This joint trend reflects a common trade-off: enhanced orientation/constraint bolsters strength but narrows large-strain compliance.

The low-frequency viscosity plateau remains comparable among formulations, while high-frequency shear-thinning diverges (Figure 7a), consistent with constraint-sensitive fast modes [81,82] in the viscoelastic spectrum; high-frequency response is governed by local segmental constraints and entanglement dynamics rather than fully relaxed network motions. Directional statistics on η* support these associations within the available subset (KH550 series + pure; Table S5a). Note that Figure 7a does not include the 570-4 wt% sample; hence, rheological comparisons involving KH570 rely on structural/mechanical/UV triangulation rather than direct η* statistics, and all controls are stated at the subset level.

log_10_(UPF) increases with improved dispersion/coverage (S_v_) and higher Γ_i_ (Figure 7b; Table S5a,b), indicating that thin yet pervasive interfacial skins extend scattering/absorption pathways at the fiber scale. Several formulations meet or surpass the practical benchmark log_10_(UPF) ≈ 1.70 (UPF = 50) [4]; replicate-level lower confidence bounds (UPF_LCB_) and UVA/UVB transmittance are reported in Table S4 under EN 13758-1 [4]/AATCC TM183 protocols [52,83]. These enhancements are consistent with classical photostabilization mechanisms of polymer matrices [83].

At 4 wt% loading, KH570 attains the highest tenacity despite a lower α/γ, consistent with an orientation-dominated reinforcement scenario where higher f(α), better dispersion, and fewer stress concentrators outweigh the benefit of higher phase fraction under anchored interfaces. Conversely, the KH550 series increases α/γ but suppresses f(α) as loading rises, aligning with a phase-fraction-dominated constraint that limits draw-induced orientation. In this compact view, KH570-4% represents the orientation-dominated endpoint (higher f(α), lower α/γ, high σ with better dispersion), whereas the KH550 loading series progressively shifts toward the phase-fraction-dominated endpoint (higher α/γ, lower f(α), increased σ but reduced ε), consistent with the two equivalent routes summarized in Figure 8b,c.

### 4.4. Interfacial Regimes: Geometric Descriptors and Structural Outcomes

We distinguish between geometric descriptors and structural outcomes. The interfacial triad (S_v_, t_i_, Γ_i_) quantifies geometry (area, effective thickness, and interfacial volume), whereas the outcome regimes describe how the structure responds: orientation-dominated versus phase-fraction-dominated. In practice, high S_v_ with smaller t_i_ tends to favor orientation-dominated responses, while lower S_v_ with larger t_i_ tends to favor phase-fraction-dominated responses. Across the filled formulations, RAF-tot normalized by the amorphous phase (φ_RAF-tot_/φ_A_) remains statistically equivalent, indicating that interfacial chemistry redistributes RAF between RAF-i and RAF-c instead of increasing the total constrained fraction (Table S2c). These outcome regimes summarize how interfacial geometry biases nucleation versus alignment pathways in semicrystalline polymer–particle systems [82,84].

Along the KH550 loading series, S_v_ decreases and t_i_ increases, and the outcome shifts toward a phase-fraction-dominated response (α/γ increases while f(α) decreases). At 4 wt%, KH570 exhibits smaller t_i_ at similar S_v_ (Table S2c) and follows an orientation-dominated response (high f(α) with low α/γ), whereas KH550 shows the complementary phase-fraction-dominated pattern (high α/γ with low f(α)). These outcome regimes rationalize the distinct strength–ductility balances seen in Figure 8b,c without implying recipe optimality.

At comparable specific interfacial area (S_v_ within 95% CIs), the functional-group chemistry of the silane coupling agent modulates adhesion versus interfacial slip, biasing the structural route. KH550 (3-aminopropyl; –NH_2_) can form stronger H-bond/Lewis acid–base interactions with PA6 amide carbonyls (and may condense with –COOH chain ends), consistent with higher interfacial anchoring/friction [30,79]; KH570 (methacryloxypropyl; –COO–) interacts more weakly and permits easier slip. Accordingly, at 4 wt% TiO_2_ and comparable S_v_, KH570 exhibits smaller t_i_ and Γ_i_ and follows an orientation-dominated response (higher f(α), lower α/γ), whereas KH550 shows larger t_i_ and Γ_i_ and a phase-fraction-dominated response (higher α/γ, lower f(α)) (Figure 5a,b; Table S2c) [44]. Cross-stage, qualitative side-evidence is consistent with this picture: the Raman amide-I band in 550-4% is stronger and slightly red-shifted; DMA storage modulus E′ peaks at 550-4%; while 570-4% attains the highest tenacity despite lower α/γ, consistent with a low-friction/thin-skin, orientation-led route. These trends are mutually coherent with the SAXS interfacial metrics (t_i_, Γ_i_), WAXS polymorphs and orientation, and DSC kinetics (see operational definitions and statistical notes of Table S5).

Spearman rank correlations (Table S5a) summarize directional associations among Γ_i_, kinetic/structural responses (ΔT_p_, −log_10_ t_1/2_, f(α)), and interfacial geometry (S_v_/t_i_), while descriptive ΔR^2^ (Table S5b) provides effect-size context under small n. Together with Porod-based surface quantification and RAF partitioning (Table S2c), the orientation- vs. phase-fraction-dominated regimes reconcile the trends observed in Figure 8a–c without invoking ranking or optimal recipes [66,69].

### 4.5. Geometry-Based Summary of RAF–Kinetics–Structure–Performance Relations

The interfacial triad (S_v_, t_i_, Γ_i_) is purely geometric, providing a transferable, reproducible way to parameterize constrained skins at filler surfaces. In practical terms, concentration mainly “turns” the geometry axis (S_v_ with induced changes in Γ_i_), while silane chemistry mainly “turns” the friction axis (effective t_i_, hence Γ_i_) at comparable S_v_. Both routes are equivalent in that they relocate samples on the same low-dimensional manifold and project onto the orientation- vs. phase-fraction-dominated outcomes captured by Figure 8b,c, while RAF_tot_/_ϕA_ remains statistically equivalent within this window.

Within the three-phase picture of semicrystalline polymers (crystal/RAF/MAF), this triad explains how a small φ_RAF-i_ can yield measurable effects when widely distributed as a sub-nanometric, high-coverage interfacial layer [1,2,59,85]. The approach builds on established SAXS invariants and Porod asymptotics to quantify specific interface area [47,48,86], with phase fractions expressed on a polymer-only basis for internal consistency.

Rather than relying on potentially misleading scatter fits at small n, we summarize directional relationships using Spearman rank correlations (exploratory, n = 4) and silane type, and we report descriptive ΔR^2^ for nested models (Figure 8a; Table S5a,b). In this compact view, Γ_i_ aligns with non-isothermal kinetics (ΔT_p_ and −log_10_t_1_/_2_), while X_c_ co-varies strongly with T_p_ on a polymer-only basis; RAF shows weak positive associations with X_c_ and α/γ. S_v_ and t_i_ show tendencies consistent with orientation and phase ratio (f(α), α/γ). Tenacity increases with T_p_ and X_c_, whereas elongation shows the opposite tendency; log_10_(UPF) trends with RAF and α/γ and exhibits near-zero association with f(α). For completeness, model-based analyses—Nakamura global fits and isoconversional methods (Kissinger/KAS)—are reported in the SI (see Figure S3 for multi-rate DSC, Figure S4 for KAS isoconversional results, Figure S5 for Kissinger plots, and Table S1 for Nakamura parameters); these reproduce the rate-dependent time-scale shifts consistent with the T_p_ trends used here, without requiring rate-wise changes in growth dimensionality within the analyzed conversion window [6,9,40,41,42,43,51].

The regime view—coverage-dominated (high S_v_, thin t_i_) vs. thickness-dominated (lower S_v_, thicker t_i_)—rationalizes concentration trends and silane-dependent pathways. At fixed loading, KH550 and KH570 can share similar S_v_ and t_i_ yet exhibit distinct kinetic–orientation signatures, indicating that interfacial chemistry modulates the activation of nucleation and alignment routes beyond geometry alone (Figure 8b,c). This is consistent with literature on polymer–nanoparticle interphases where interfacial layer thickness and interaction strength co-govern dynamics and properties [34].

Coarse-grained MD (Figure S11) illustrates stronger interfacial adhesion and more upright chain alignment near KH550-modified surfaces compared with the passivated KH570 surfaces, qualitatively consistent with the kinetic shifts inferred from DSC. We emphasize that these simulations visualize plausible local motifs; absolute temperatures depend on mapping and potential simplifications.

Overall, the (S_v_, t_i_, Γ_i_) triad, coupled with small-sample-robust statistics, offers a reproducible and transferable protocol to connect interface-resolved RAF with kinetics, structure, and performance across polymer–nanoparticle fiber systems [5]. Building on the interphase perspective emphasized by Huang et al. [34], our study quantifies and reproducibly partitions RAF into interfacial and crystal-adjacent components. By integrating SAXS invariants and DSC kinetics within fiber systems, we operationalize the interphase concept into transferable descriptors (S_v_, t_i_, Γ_i_), thereby providing a practical, cross-scale basis for design.

For operational clarity, we use a minimal two-readout notion of “compatibility”: (i) nucleation-compatibility, summarized by ΔT_p_ (and apparent E_a_ from KAS/Kissinger) evaluated at comparable S_v_ (or expressed per unit S_v_ when appropriate); and (ii) constraint-compatibility, summarized by Γ_i_ and t_i_ (=Γ_i_/S_v_). We employ these strictly as operational readouts, not as theory claims; full operational definitions and statistical notes are provided in Table S5 notes.

## 5. Conclusions

We established an interface-to-performance link for PA6/TiO_2_ fibers that is consistent across kinetics, structure, and properties. On a polymer-only basis, the total rigid amorphous fraction remains approximately conserved, while its allocation redistributes from crystal-adjacent RAF to filler-proximal interfacial RAF, thereby addressing transferable interphase quantification at the fiber scale. To summarize interfacial participation without over-emphasizing thickness magnitudes, we use a geometry-aware pair (S_v_, Γ_i_) derived from SAXS invariants: specific interfacial area and the interfacial RAF volume within the composite—with t_i_ treated as an effective descriptor.

- Γ_i_ correlates with crystallization metrics (higher T_p_, longer t1/2), while α/γ and f(α) provide orthogonal structural coordinates.
- “Compatibility” is described operationally as nucleation-compatibility and constraint-compatibility, consistent with RAF views.
- KH550 and KH570 yield distinct coupling routes (phase-fraction–dominated vs. orientation-dominated).
- Performance changes arise from redistribution and pathway choice, not from a net increase in RAF.

The (S_v_, Γ_i_) descriptors and the associated workflow based on SAXS invariant together with WAXS and DSC provide a transferable, reproducible basis for interphase-aware design of polymer–nanoparticle fibers, thereby closing the kinetics–structure–property loop articulated in the Introduction.

This work examined three compositions including pure PA6 (0, 1.6, and 4.0 wt% TiO_2_) in a single PA6 matrix under a fixed melt-spinning window (temperature, take-up speed, DR ≈ 3.0) and two coupling agents (KH550 vs. KH570 at a representative loading); thus composition–processing interactions are bounded by this design. Several selected datasets were acquired as single-run; robustness checks and cross-method closure were applied. The SAXS-derived interphase metrics (S_v_, Γ_i_, t_i_) rely on Porod invariants after Bragg subtraction and a polymer-only basis, whose sensitivity to model choices and particle polydispersity warrants further tests (e.g., contrast variation, tomography). Application-side validation (operando/in situ crystallization, long-term mechanical/UV durability, and laundering stability for UPF) was outside the present scope. Future work will broaden filler fractions and surface chemistries, map the spinning parameter space (T, ω, DR) using designed experiments, include independent replicates (n ≥ 3) and inter-lab checks, and integrate MD/DFT-guided interface design with process optimization to generalize the interphase-geometry–performance framework across polymer–nanoparticle fiber systems.

## Figures and Tables

**Table 1:** Fiber samples used for quantitative analysis and their preparation parameters.

Sample ID	Silane Coupling Agent	TiO_2_ Content (wt%)	Draw Ratio
pure PA6	None	0	3.0
PA6-1.6 wt% TiO_2_-KH550	KH550	1.6	3.0
PA6-4.0 wt% TiO_2_-KH550	KH550	4.0	3.0
PA6-4.0 wt% TiO_2_-KH570	KH570	4.0	3.0

Sample IDs (e.g., 550-1.6%, 570-4%) are shorthand notations used consistently in figures and tables to denote PA6/TiO_2_ fibers with specified TiO_2_ content (wt%) and silane coupling agents (KH550 or KH570).

## Data Availability

The datasets generated and analyzed during this study are available in the Zenodo repository at DOI: 10.5281/zenodo.16983099 (all versions), with Version v1.0 archived at DOI: 10.5281/zenodo.16983100.

## Supplementary Materials

The following supporting information can be downloaded at: https://www.mdpi.com/article/10.3390/polym17182551/s1, The following supporting information can be provided upon acceptance. Figure S1: SEM micrographs of melt-spun PA6/TiO_2_ fibers at representative loadings; Figure S2: Full-range Raman spectra (100–1800 cm^−1^) of injection-molded PA6/TiO_2_ composites (KH550/KH570 series); Figure S3: Full non-isothermal DSC cooling curves at β = 5–40 °C·min^−1^; Figure S4: Per-conversion KAS regressions (ln[β/T_x_^2^] vs. 1/T_x_) at X = 0.30–0.55; Figure S5: Kissinger regressions (ln[β/T_p_^2^] vs. 1/T_p_) and fitted E_k_; Figure S6: Raw SAXS profiles and Bragg-peak subtraction used for Porod invariants; Figure S7: Interfacial rigid amorphous fraction (φRAF-i) and normalized total RAF (RAFtot/φA, polymer-only basis) with 95% CIs and TOST equivalence assessment; Figure S8: Robustness of the Hermans factor extraction at the PA6 α(200) reflection; Figure S9: Representative peak deconvolution of the WAXS pattern for the 550-1.6% sample; Figure S10: Azimuthal profile at PA6 α(200) for Hermans factor evaluation; Figure S11: Coarse-grained MD snapshots and DSC-like heat-flow curves (−dU/dT); Figure S12: SEM–EDS analyses confirming fibrous morphology and TiO_2_ dispersion at 1.8% and 6% loadings. Figure S13. Robustness check of pure PA6 DSC curve. The 165 °C jitter is due to overlap, not data loss; raw and processed curves give consistent ΔH_c_ and T_p_. Table S1: Nakamura (θ*) parameters and uncertainties under non-isothermal cooling; Table S2: SAXS results panel—(a) Porod invariants by q-range; (b) phase fractions (φc, φRAF-c, φRAF-i, φMAF) on a polymer-only basis; (c) interfacial-layer metrics (Sv, Γi, ti); (d) low-q baselines and increments (ΔQ_1_) used to fix φRAF-i; (e) master summary with 95% CIs and absolute scale factor α; Table S3: Robustness of the α(200) Hermans factor; Table S4: Ultraviolet protection metrics (UPF, T(UVA), T(UVB)) for woven fabrics; Table S5a,b: Partial correlations and descriptive ΔR^2^ linking interfacial metrics (Γi, Sv, ti) to proximal responses (ΔTp, t1/2, f(α), η*).
