# Supplementary material for: Interphase-Resolved Performance in PA6/TiO2 Nanocomposite Fibers: Four-Phase Geometry Linking Structure to Mechanical and UV Protection
Source: Polymers (Basel). 2025 Sep 21;17(18):2551. doi: 10.3390/polym17182551 (PMC12473844; doi:10.3390/polym17182551)
Supplement: Supplementary file 1 [file polymers-17-02551-s001.zip › polymers-3870679_Supplementary_Materials.docx.pdf]

Interphase-Resolved Performance in PA6/TiO_2 Nanocomposite Fibers: Four-Phase Geometry Linking Structure to Mechanical and UV Protection

Hailong Yu[1], Ping Liu[1], Xiaohuan Ji[2], Xiaoze Jiang[1,*] and Bin Sun[1,*]

1 State Key Laboratory of Advanced Fiber Materials, College of Materials Science and Engineering, Donghua University, Shanghai 201620, China
2 Shaoxing Huiqun New Material Technology Co., Ltd., Shaoxing 312000, China
* Correspondence: xiaozejiang@dhu.edu.cn; sunbin@dhu.edu.cn

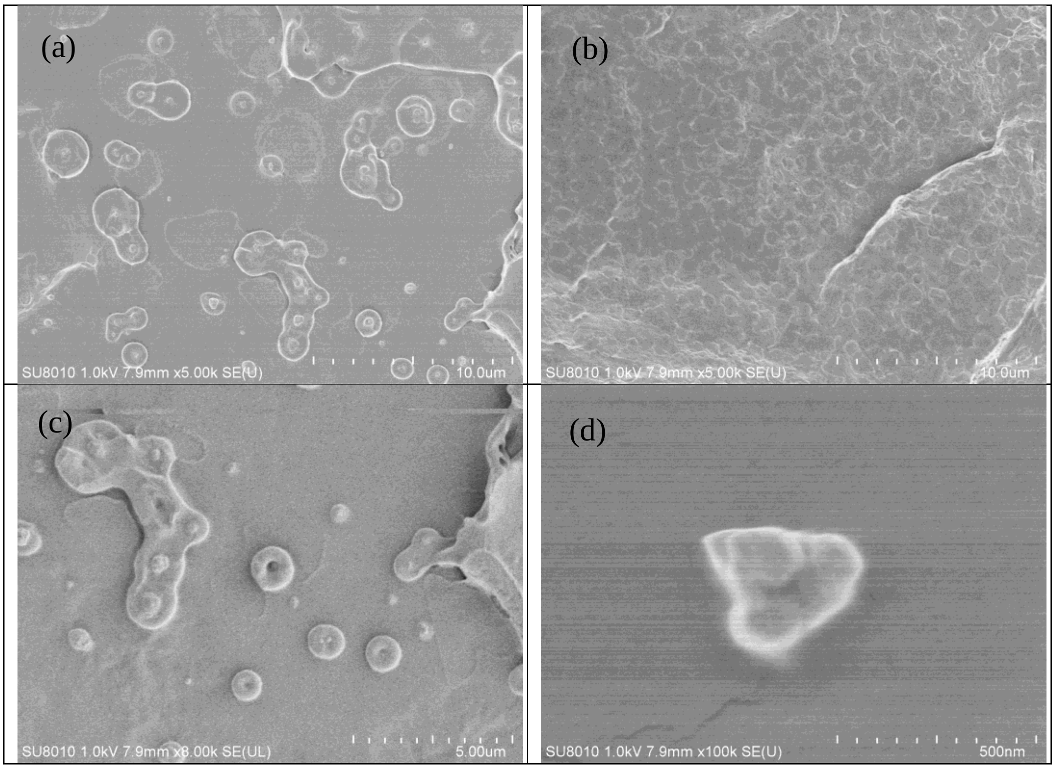

Figure S1. SEM micrographs of melt-spun PA6/TiO_2 fibers at representative loadings. (a) 1.8 wt% (10 μm): higher particle density with uniform distribution; ring-like features correspond to TiO_2 cores surrounded by the PA6 matrix or an interfacial phase. (b) 4.0 wt% (10 μm): increased particle density compared with (a), with some local particle adjacency but no continuous agglomerated domains. (c) 1.8 wt% (5 μm): clearer ring-like morphology at higher magnification. (d) 1.8 wt% (500 nm): individual TiO_2 particle or TiO_2 particle with a tightly bound interfacial layer; the doughnut-like morphologies in (a) and (c) correspond to such cores with surrounding phases. EDS analysis of the 6.0 wt% sample indicated Ti ≈ 7.9 wt% (2.22 at.%), consistent with nominal filler loading. All images were obtained from cryo-fractured fibers and are provided for qualitative assessment of dispersion; quantitative dispersion metrics are reported in the main text. Samples correspond to related melt-spun PA6/TiO_2 fibers prepared using the same processing route as in this work. In (b), 4.0 wt% TiO_2 fibers show isolated particle adjacency without formation of continuous agglomerated domains, consistent with the description in the main text.

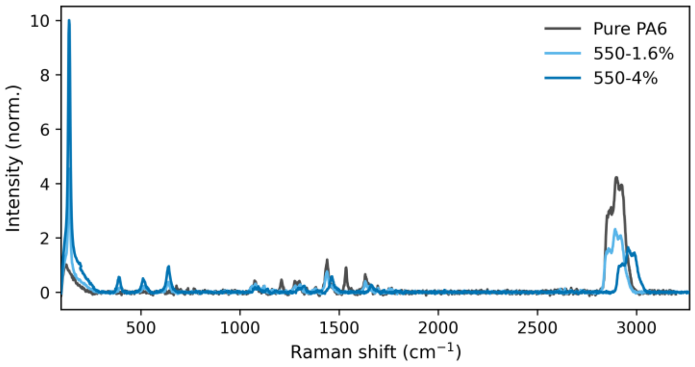

Figure S2. Full-range Raman spectra (100–1800 cm^{-1}) of injection-molded PA6/TiO_2 composites with different TiO_2 loadings: KH550 series (0, 1.6, and 4 wt%) and KH570 series (4 wt%). Spectra were normalized to the 95th percentile intensity over the plotted range. The 100–700 cm^{-1} region, corresponding to crystalline lattice and skeletal vibrations, is shown in Figure 2b of the main text, and the amide I region (1600–1700 cm^{-1}) is magnified in its inset. Minor differences in the high-wavenumber region (~2800–3200 cm^{-1}) are attributed to variations in C–H stretching modes and do not influence the structural or crystallization analyses presented in this work.

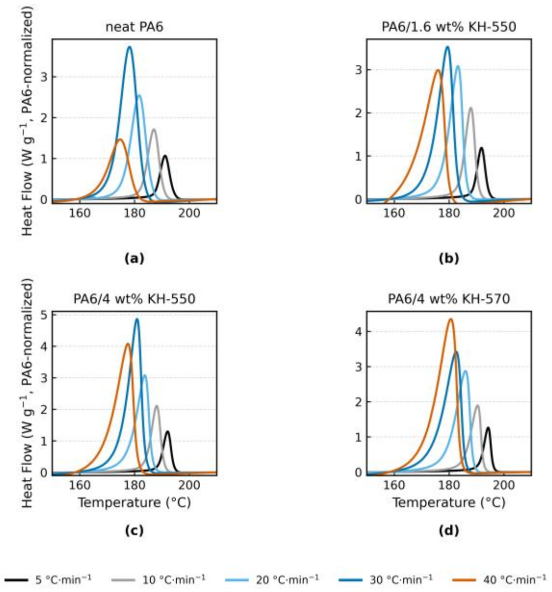

Figure S3. Full non-isothermal DSC cooling curves for each formulation (panel, 2×2). For every sample, curves at cooling rates β = 5, 10, 20, 30, and 40 °C·min^{-1} are overlaid. Temperature axis is fixed to 150–210 °C for cross-comparison. Heat flow is PA6-normalized (W g^{-1}) after TiO_2 blank correction; exotherm is plotted upward. (Colors map to cooling rates consistently across panels: de5–de40.)

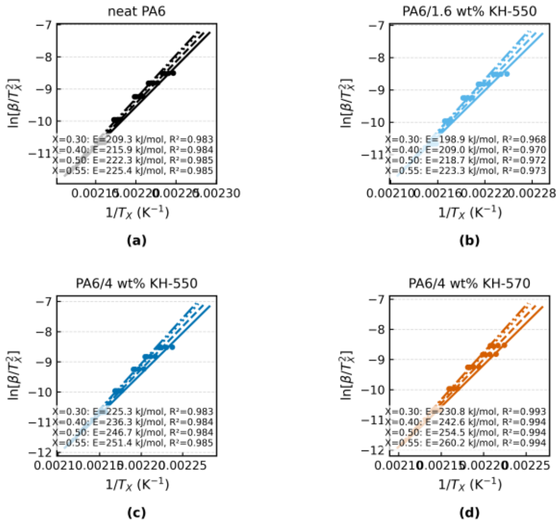

Figure S4. Per-conversion KAS (multi-point) regressions for each formulation (panel, 2×2): $\ln[\beta/T_x^2]$ versus $1/T_x$ at X = 0.30, 0.40, 0.50, and 0.55 using β = 5–40 °C·min⁻¹ (≥ 3 rates per X). Symbols are experimental points; lines are least-squares fits. Line-style mapping (if the legend is omitted): solid = X = 0.30, dashed = X = 0.40, dotted = X = 0.50, dash-dot = X = 0.55.

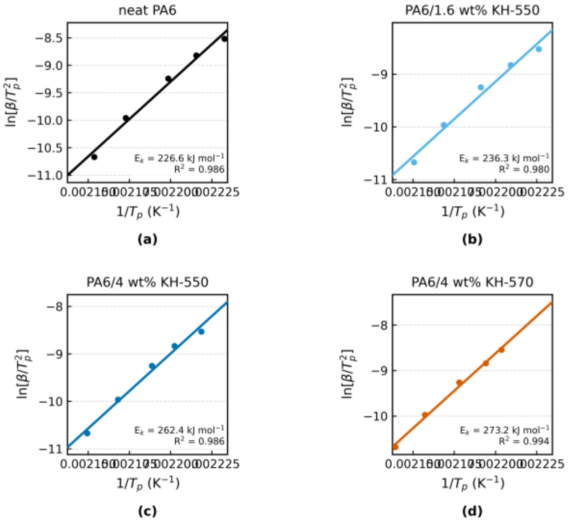

Figure S5. Kissinger (single-point) regressions for each formulation: $\ln[\beta/T_p^2]$ versus $1/T_p$ at β = 5–40 °C·min⁻¹. The fitted slope yields E_k (kJ·mol⁻¹); the corresponding R^2 values are annotated in each plot.

Table S1. Nakamura (θ*) parameters and uncertainties under non-isothermal cooling. Fits over $0.20 \le X_t \le 0.60$ with E fixed from the isoconversional closure; $\hat{\alpha}$ obtained from ln K0* ~ $\hat{\alpha}$ ln β. Values are estimate [95% CI].

Sample (composition)	E (kJ·mol⁻¹)	n [95% CI]	$\hat{\alpha}$ [95% CI]	K_0* (de5)	K_0* (de10)	K_0* (de20)	K_0* (de30)	K_0* (de40)
Pure PA6	208.19	3.147 [3.063–3.262]	2.096 [2.087–2.104]	3.03E−03	1.04E−02	4.34E−02	1.07E−01	2.51E−01
550-1.6%	202.94	2.871 [2.759–3.003]	2.121 [2.110–2.132]	3.28E−03	1.10E−02	4.46E−02	1.14E−01	2.98E−01
550-4%	229.60	2.762 [2.670–2.877]	2.103 [2.094–2.112]	3.44E−03	1.21E−02	5.30E−02	1.19E−01	2.97E−01
570-4%	237.44	2.360 [2.297–2.431]	2.108 [2.099–2.118]	3.41E−03	1.28E−02	5.36E−02	1.46E−01	2.67E−01

Notes: 95% confidence intervals for n and $\hat{\alpha}$ were obtained via nonparametric bootstrap (B = 500) within 0.20–0.60 conversion. K_0* values are per-rate point estimates (units s^{-1} if θ* is in seconds). E values are fixed from the independent isoconversional analysis used in the closure procedure.

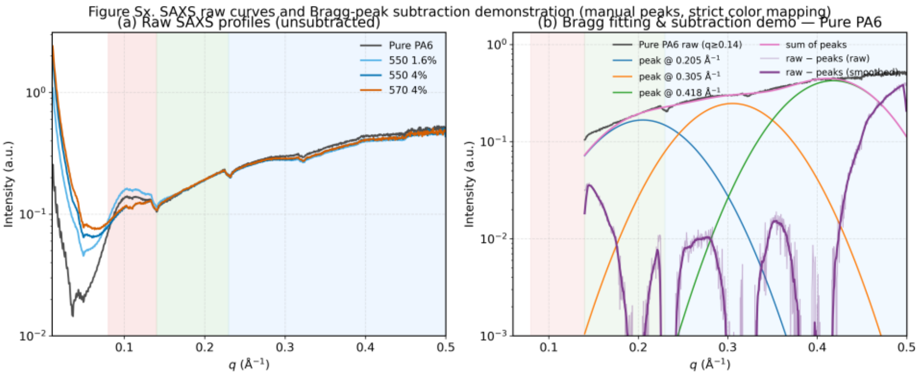

Figure S6. (a) Raw SAXS intensity profiles of PA6/TiO_2 fibers, corresponding to the samples in Figure 4a, showing the q-ranges used for Porod invariant integration (Table S2). (b) Example of Bragg-peak fitting and subtraction for Pure PA6, starting from q = 0.14 $Å^{-1}$, as used in the calculation of Q values. Colored curves indicate manually selected Bragg peaks, the magenta curve is their sum, and the purple curves are the residual profiles (raw – peaks, raw and smoothed).

Table S2. SAXS-derived panels (bootstrap 95% CIs; polymer-only basis).

This supplementary table compiles five coordinated panels: (a) Porod-invariant integrals within predefined q-bands; (b) phase fractions obtained with a fixed pipeline, in which the low-q difference method is used to fix (ϕ_RAF-i) (relative to neat PA6), followed by Q23-based partitioning of (ϕ_RAF-c) and (ϕ_MAF); (c) interfacial metrics used for interpretation (S_v, Γ_i, t_i); (d) low-q baselines and increments that determine (ϕ_RAF-i);

and (e) a compact master summary with 95% confidence intervals and the absolute-scale factor α. All phase fractions are reported on a polymer-only basis.

(a) Porod invariants (Q) for different q-ranges

Sample	Q1	Q2	Q3	Q_total
550 4%	1.010×10^{-4}	7.285×10^{-6}	1.513×10^{-3}	1.621×10^{-3}
550 1.6%	1.187×10^{-4}	7.851×10^{-6}	1.528×10^{-3}	1.654×10^{-3}
Pure PA6	9.969×10^{-5}	7.553×10^{-6}	1.575×10^{-3}	1.683×10^{-3}
570 4%	1.049×10^{-4}	1.024×10^{-5}	1.021×10^{-3}	1.136×10^{-3}

(b) Panel b. Phase fractions on polymer-only basis (means [95% CI]; φ_c, RAF-i, RAF-c, MAF).

Sample	φ_c (DSC)	RAF-i (95% CI)	RAF-c (95% CI)	MAF (95% CI)	RAF_tot (95% CI)	RAF_tot/φ_A (95% CI)	α (alpha)	sum_check
550–1.6% TiO_2	0.25 [0.25, 0.25]	0.00231 [0.00188, 0.00273]	0.23163 [0.23163, 0.23163]	0.51606 [0.51565, 0.51649]	0.23394 [0.23351, 0.23436]	0.312 [0.311, 0.312]	3.56993	1
570–4% TiO_2	0.2695 [0.2695, 0.2695]	7e-05 [0, 0.00022]	0.22598 [0.22598, 0.22598]	0.50441 [0.50426, 0.50448]	0.22605 [0.22598, 0.2262]	0.309 [0.309, 0.31]	3.56993	0.99996
550–4% TiO_2	0.2699 [0.2699, 0.2699]	0.00025 [0.0001, 0.00041]	0.2304 [0.2304, 0.2304]	0.49943 [0.49927, 0.49958]	0.23065 [0.2305, 0.23081]	0.316 [0.316, 0.316]	3.56993	0.99998
Pure PA6	0.2153 [0.2153, 0.2153]	0 [0, 0]	0.2354 [0.2354, 0.2354]	0.5493 [0.5493, 0.5493]	0.2354 [0.2354, 0.2354]	0.3 [0.3, 0.3]	3.56993	1

(c) Interfacial metrics.

Sample_Desc	phi_f	RAF_i_mean	t_i_nm	S_v_filler_nm^-1	S_v_comp_nm^-1	Gamma_i
550–1.6% TiO_2	0.016	0.00231	0.0123	0.161	0.002273	0.185
570–4% TiO_2	0.04	7e-05	0.0034	0.152	6.72e-05	0.0197
550–4% TiO_2	0.04	0.00025	0.00304	0.152	0.00024	0.0791
Pure PA6	0	0	0.149	0		

(d) Low-q baselines and increments (difference method)

Sample	q12_used	Q1_pure_interp	Q1_sample	ΔQ1 (95% CI)
550–1.6% TiO_2	0.14	0.0390983	0.073491	0.0343927 [0.0329633, 0.0358668]
570–4% TiO_2	0.14	0.0390983	0.0930282	0.0539299 [0.0511145, 0.0566577]
550–4% TiO_2	0.14	0.0390983	0.109777	0.0706788 [0.0669014, 0.0741305]
Pure PA6	0.14	0.0390983	0.0390983	0 [0, 0]

(e) Summary with 95% CIs and absolute-scale factor α (bootstrap; φ_A-scale = 1).

Sample	α (alpha)	φ_A (abs)	RAF-i (95% CI)	RAF-c (95% CI)	MAF (95% CI)	RAF_tot (95% CI)	RAF_tot/φ_A (95% CI)
550–1.6% TiO_2	3.56993	0.75 [0.75, 0.75]	0.00231 [0.00188, 0.00273]	0.23163 [0.23163, 0.23163]	0.51606 [0.51565, 0.51649]	0.23394 [0.23351, 0.23436]	0.312 [0.311, 0.312]
570–4% TiO_2	3.56993	0.7305 [0.7305, 0.7305]	7e-05 [0, 0.00022]	0.22598 [0.22598, 0.22598]	0.50441 [0.50426, 0.50448]	0.22605 [0.22598, 0.2262]	0.309 [0.309, 0.31]
550–4% TiO_2	3.56993	0.7301 [0.7301, 0.7301]	0.00025 [0.0001, 0.00041]	0.2304 [0.2304, 0.2304]	0.49943 [0.49927, 0.49958]	0.23065 [0.2305, 0.23081]	0.316 [0.316, 0.316]
Pure PA6	3.56993	0.7847 [0.7847, 0.7847]	0 [0, 0]	0.2354 [0.2354, 0.2354]	0.5493 [0.5493, 0.5493]	0.2354 [0.2354, 0.2354]	0.3 [0.3, 0.3]

Notes: Definitions. ϕ_c = crystalline fraction; ϕ_RAF-c = crystal-adjacent rigid amorphous fraction; ϕ_RAF-i = interfacial rigid amorphous fraction; ϕ_MAF = mobile amorphous fraction; ϕ_A = amorphous fraction = 1 − ϕ_c; ϕ_RAF-tot = ϕ_RAF-c + ϕ_RAF-i. Polymer-only basis. Unless noted otherwise, all phase fractions are normalized to the polymer internal volume (i.e., polymer-only basis).

Uncertainty. 95% confidence intervals (CIs) are percentile bootstrap intervals (B = 1000 by default). Equivalence testing. For ϕ_RAF-tot/ϕ_A, two one-sided tests (TOST) with an equivalence margin of ±0.01 are used in the main text. Absolute scaling. α (alpha_mean) is the absolute intensity scale factor carried consistently across panels; where listed, the same α is used for transparency. Rounding. Values are rounded to three or four significant digits for readability; computations are performed in double precision. Bragg handling & preprocessing. All samples share the same preprocessing (peak masking/de-Bragg, background convention, q-band edges).

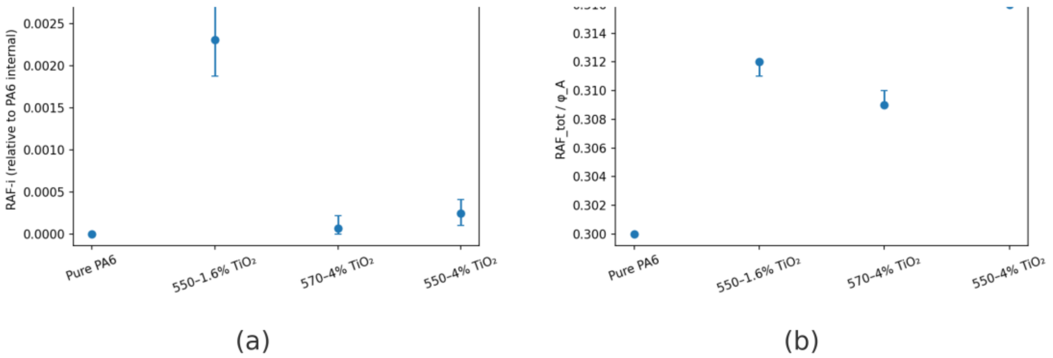

Figure S7. Interfacial vs. total rigid amorphous fractions from SAXS (polymer-only basis; 95% CIs).(a) ϕ_RAF-i for each formulation, obtained by the low-q difference method ($\Delta Q_1 = Q_1$,Sample − Q_1,Pure) and then fixed in the phase fit; neat PA6 is constrained to ϕ_RAF-i = 0. (b) Total rigid amorphous fraction normalized by the amorphous content, RAF_tot/ϕ_A = (ϕ_RAF-c + ϕ_RAF-i)/ϕ_A. Markers are means; error bars are 95% bootstrap confidence intervals (B = 1000). Among the three TiO_2-filled samples, RAF_tot/ϕ_A falls within a narrow band and is statistical-ly equivalent under a ±0.01 TOST margin, whereas neat PA6 shows the expected lower baseline.

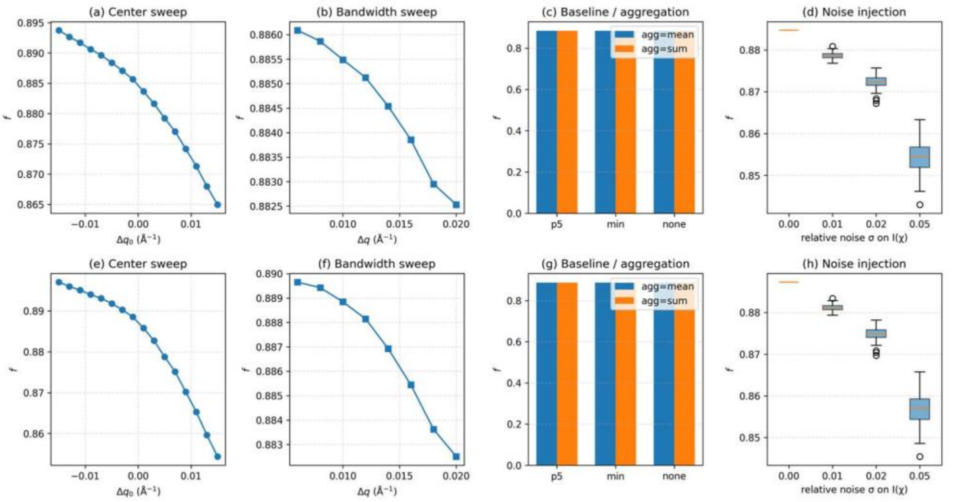

Figure S8. Robustness of the Hermans factor extraction at the PA6 α(200) reflection. Panels (a–d): KH570–4 wt%; panels (e–h): KH550–4 wt% (top row = KH570, bottom row = KH550). For each sample: (a,e) sensitivity to the band center q_0 (reported as offsets from the canonical q_0); (b,f) sensitivity to the half-bandwidth Δq; (c,g) comparison of baseline modes (“none”, “p5”, “min”) and radial aggregation (“mean” vs “sum”); (d,h) robustness to injected zero-mean Gaussian noise with σ =

η·median[I(χ)]. Canonical settings (for bars labeled "canonical"): q_0 from the batch script auto-located α(200) peak center; Δq = 0.020 $Å^{-1}$ (unless stated otherwise); baseline = none; aggregation = sum; φ = 90°; polarization = 0.95; exposure-scaled air subtraction; χ-sector exclusion −180° to −160° and 0° to 20°; γ-band TiO_2 notches applied. These checks indicate that the qualitative conclusions are insensitive to processing choices.

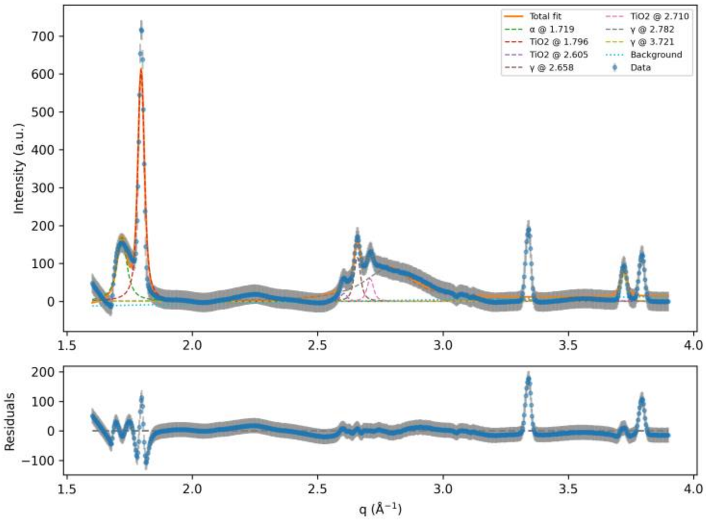

Figure S9 Representative peak deconvolution of the baseline-corrected WAXS pattern for the 550–1.6 wt% TiO_2 sample. The PA6 crystalline peaks of the α-form [e.g., α(200), α(002/202), α(210)] and γ-form [e.g., γ(001), γ(201/101), γ(210)] are modeled with pseudo-Voigt functions under a common baseline; sharp TiO_2 peaks are treated as separate narrow components or masked when needed. Fit residuals are shown to illustrate quality. The resulting α/γ ratio and $D\gamma^{(rep)}$ are consistent with the summary in Figure 5a. Processing steps (baseline, masking, and bounds) follow those used for all samples.

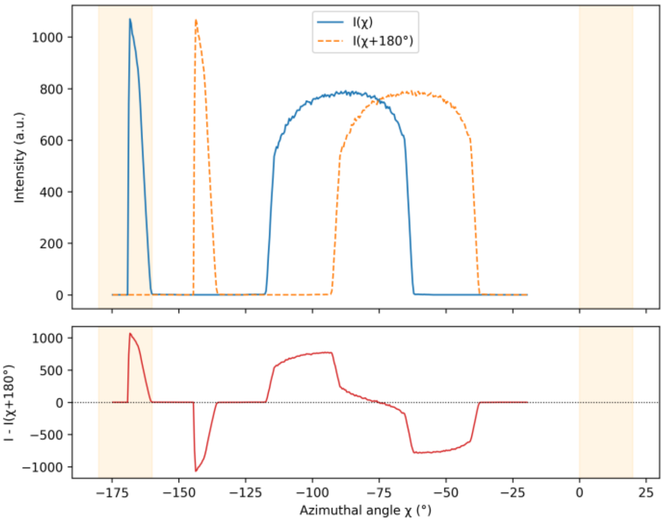

Figure S10. Representative azimuthal profile at the PA6 α(200) reflection for the 570–4% fiber. Intensity I(χ) (solid) and the 180°-shifted I(χ+180°) (dashed) nearly overlap after excluding instrument-affected χ sectors (−180° to −160°, 0° to 20°; shaded), validating a φ offset of 90°. The q-band was centered at the α(200) peak with the final half-width used in the analysis; polarization and

solid-angle corrections and exposure-scaled air subtraction were applied. The resulting Hermans factor fα from this band is consistent with the bar chart in the main text.

Table S3. Robustness of the α(200) Hermans factor (fα)

(a) Effect of q-band half-width (dq) on fα at PA6 α(200) (sample 570–4%). Values are mean ± s.d. from N = 300 Monte-Carlo realizations; other processing as in the main text.

dq (Å^{-1})	fα	σ_MC
0.006	0.905027	0.000762
0.008	0.905040	0.000763
0.010	0.904980	0.000764
0.012	0.904980	0.000764
0.015	0.904968	0.000764
0.020	0.904965	0.000763
0.030	0.904719	0.000765

(b) Effect of band-center q_0 on fα (excluded-sector evaluation). q_0 is scanned around 1.7168 Å^{-1}; other processing as in the main text.

q0 (Å^{-1})	fα (excluded)
1.701800	0.915981
1.706800	0.913482
1.711800	0.911127
1.716800	0.908556
1.721800	0.905935
1.726800	0.903549
1.731800	0.901122

C) Noise-injection robustness at α(200). Zero-mean Gaussian noise with $\sigma = \eta \cdot \text{median}(I)$ was added to I(χ) and fα was re-evaluated (N = 300 trials). Values report mean ± s.d. All values refer to α(200)-based Hermans factor.

η	fα	s.d.
0.01	0.922	0.0008
0.02	0.914	0.0015
0.05	0.893	0.0037
0.10	0.859	0.0066

Note: WAXS processing included solid-angle and polarization correction (0.95), exposure-scaled air subtraction, union mask, χ-sector exclusion (−180:−160°, 0:20°), and a light p5 baseline. All values refer to α(200)-based Hermans factor.

Table S4. Ultraviolet protection metrics for pure PA6 and TiO_2-filled woven fabrics. Values are means over replicate scans. UPF_LCB denotes the 95% one-sided t-based lower confidence bound. Spectral range: 290–400 nm; solar irradiance profile: EN 13758-1:2002.

Sample	Linear density (dtex)	UPF (mean ± SD)	UPF_LCB (95%)	n	T(UVA) % (mean ± SD)	T(UVB) % (mean ± SD)
pure PA6	45	5.02 ± 0.20	4.78	4	32.81 ± 1.34	17.39 ± 0.66

550–1.6%	82	666.33 ± 30.91	636.86	5	2.04 ± 0.12	0.05 ± 0.00
550–4%	61	246.61 ± 18.98	224.28	4	2.38 ± 0.08	0.27 ± 0.03
570–4%	50	81.30 ± 4.08	77.41	5	3.11 ± 0.20	1.12 ± 0.06

Notes. UPF was computed per EN 13758-1/AATCC TM183 from 290–400 nm spectra. UPF_LCB = mean − $t_{0.95}$,df × SD/ √ n (one-sided). Percent values are spectral transmittance means ± SD (%).

Table S5a. Partial Spearman ρ(y, x ∣ wt%, KH570) — Proximal responses (exact permutation p-values).

y (proximal response)	x (predictor)	controls	rho_partial	p_exact	n
ΔT_p (°C) vs neat	Γ_i	wt_pct, silane_KH570	-1.000	0.042	4
ΔT_p (°C) vs neat	S_v (nm^-1)	wt_pct, silane_KH570	0.500	0.667	3
ΔT_p (°C) vs neat	t_i (nm)	wt_pct, silane_KH570	-1.000	0.333	3
−log10(t_1/2)	Γ_i	wt_pct, silane_KH570	-1.000	0.042	4
−log10(t_1/2)	S_v (nm^-1)	wt_pct, silane_KH570	0.500	1.000	3
−log10(t_1/2)	t_i (nm)	wt_pct, silane_KH570	-1.000	0.333	3
f_alpha (Hermans, α(200))	Γ_i	wt_pct, silane_KH570	-1.000	0.042	4
f_alpha (Hermans, α(200))	S_v (nm^-1)	wt_pct, silane_KH570	-0.500	1.000	3
f_alpha (Hermans, α(200))	t_i (nm)	wt_pct, silane_KH570	1.000	0.333	3
η* @ 100 rad/s (Pa·s)	Γ_i	wt_pct, silane_KH570	1.000	0.333	3
η* @ 100 rad/s (Pa·s)	S_v (nm^-1)	wt_pct, silane_KH570	NA	NA	2
η* @ 100 rad/s (Pa·s)	t_i (nm)	wt_pct, silane_KH570	NA	NA	2

Table S5b. Descriptive ΔR^2 when adding Γ_i to y ~ wt% + KH570 — Proximal responses (no hypothesis tests at small n).

y (proximal response)	R²_small (wt%+KH570)	R²_big (+Γ_i)	+ΔR²	n
ΔT_p (°C) vs neat	0.995	1.000	0.005	4

−log10(t_1/2)	0.950	1.000	0.050	4
f_alpha (Hermans, α(200))	0.961	1.000	0.039	4
η* @ 100 rad/s (Pa·s)	0.368	1.000	0.632	3

Notes: Pure PA6 lacks defined S_v and t_i; for those predictors, analysis uses the filled subset ($n \leqslant 3$). η* is available only for KH550 series and the neat sample, hence the KH570 dummy is zero in that subset. Exact permutation p-values are reported to avoid over-interpretation at small n; ΔR^2 are descriptive only.

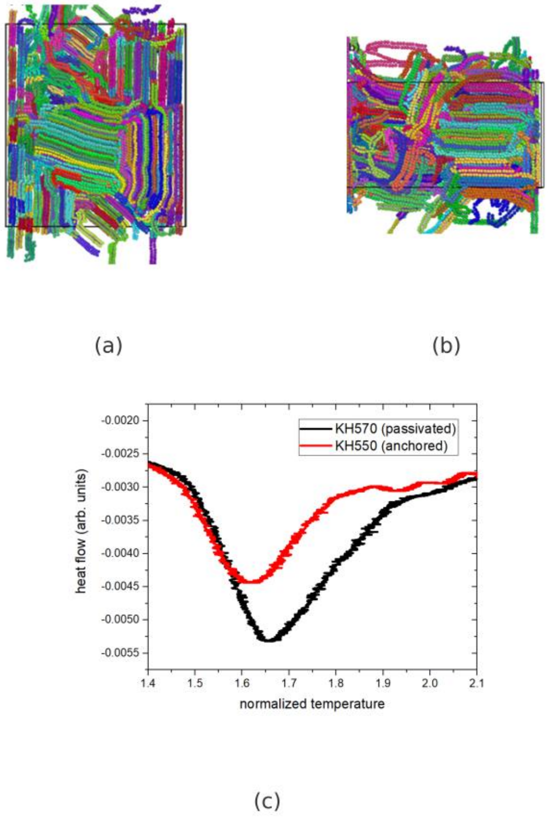

Figure S11. Coarse-grained MD snapshots and simulated heat-flow curves. (a) Representative snapshot near a KH570-passivated TiO_2 wall. (b) Representative snapshot near a KH550-anchored TiO_2 wall, where polymer chains exhibit stronger interfacial adhesion and more upright alignment. Colors distinguish chains only; the simulation box is outlined in black. (c) DSC-like heat-flow (arbitrary units) vs temperature obtained from −dU/dT of the CG simulations. Black: KH570-passivated; red: KH550-anchored. The difference in peak temperature indicates distinct crystallization temperatures, consistent with experimental trends. The CG simulations are intended to qualitatively compare interfacial trends rather than provide absolute predictions.

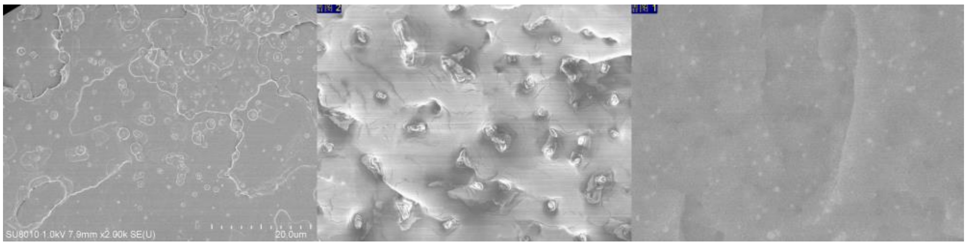

(a) (b) (c)

Figure S12. (a) Low-magnification SEM image (2k×) confirming the fibrous morphology of melt-spun PA6/TiO_2 composites. (b–c) Representative SEM–EDS analyses of TiO_2-modified fibers at nominal 6 wt% and 1.8 wt% loadings, respectively. Ti and O signals coincide with bright-contrast particle regions, and measured Ti levels (7.9 wt% and 1.0 wt%) are consistent with nominal formulations. These data corroborate effective dispersion enhanced by silane modification, while also indicating occasional local adjacency at higher loading without the formation of extended agglomerated domains.

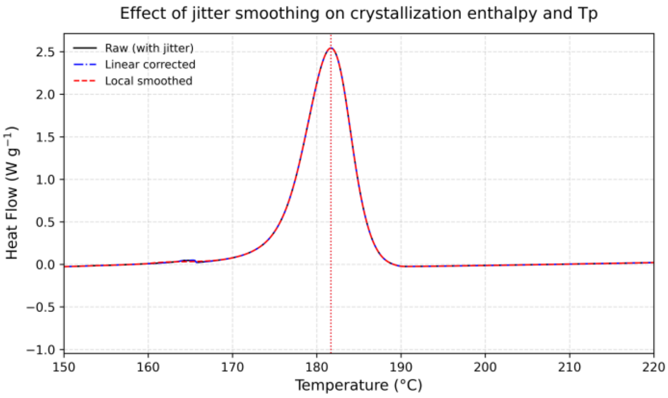

Method	ΔHc (J/g)	Tp (°C)
Raw (jitter)	54.97	181.68
Linear	54.97	181.68
Local smoothed	54.99	181.68

Figure S13. Robustness check of the pure PA6 DSC crystallization curve. The apparent interruption of the PA6 trace near 165 °C in Figure 3 originates from overlap with other sample curves (550–1.6%, 550–4%, and 570–4%), not from data truncation. The raw PA6 data are continuous, and a comparison of raw, linear baseline–corrected, and locally smoothed curves confirms that the minor jitter in this region does not affect crystallization enthalpy (ΔH_c = 54.97 vs 54.99 J/g) or peak temperature (T_P = 181.68 °C).
